# Exploration of Novel Indole Compounds with Potential Activity Against Breast Cancer: Synthesis, Characterization and Anti-Cancer Activity Evaluation

**DOI:** 10.3390/ph19030418

**Published:** 2026-03-04

**Authors:** Eid E. Salama, Ashtar A. Alrayes, Saad Alrashdi, Ahmed T. A. Boraei, Nagwa I. Ahmed, Salah Eid, Karam S. El-Nasser, Haitham Kalil, Ahmed A. M. Sarhan

**Affiliations:** 1 Department of Chemistry, College of Science, Jouf University, Sakaka 72341, Aljouf, Saudi Arabia; aarayes@ju.edu.sa (A.A.A.); saalrashdi@ju.edu.sa (S.A.);; 2 Department of Chemistry, Faculty of Science, Suez Canal University, Ismailia 41522, Egypt; 3 Internal Medicine Department, Faculty of Medicine, Suez University, Suez 43511, Egypt; 4 Department of Chemistry & Biochemistry, California Polytechnic State University, San Luis Obispo, CA 93407, USA; 5 Department of Chemistry, Faculty of Science, Arish University, Al-Arish 45511, Egypt

**Keywords:** indolyl-1,2,4-triazoles, synthesis, anticancer, MCF-7, HepG2

## Abstract

**Background/Objectives:** Cancer remains one of the most significant challenges in modern medicine, requiring the continuous development of novel molecular scaffolds with anticancer potential that act through multiple pathways. Heterocyclic compounds incorporating indole, triazole, oxadiazole, and thiadiazine motifs have attracted considerable attention due to their diverse pharmacological activities. This study aimed to design, synthesize, and evaluate new hybrid heterocyclic systems, including 1,2,4-triazole, 1,3,4-oxadiazole, and thiadiazine motifs, targeting liver and breast cancer. **Methods:** A series of indolyl-based heterocyclic compounds was synthesized using efficient and environmentally friendly protocols. Indolyl-triazol-thiadiazin-6-ol **5** was prepared via solvent-free fusion of esters **2** and **3** or the corresponding acid **4**. Oxadiazole derivatives were produced by reacting hydrazide intermediates with carbon disulfide. Triazole derivatives were synthesized via cylization of thiosemicarbazide **9** in aqueous KOH (4.0 N). Structural characterization was performed using Fourier Transform InfraRed (FTIR), ^1^H and ^13^C NMR spectroscopy, and electron impact mass spectrometry (EIMS). Cytotoxic activity was evaluated against liver and breast cancer cell lines, and VEGFR-2 kinase inhibition was assessed for selected derivatives. **Results:** The synthesized compounds demonstrated notable cytotoxicity activity, with compounds **4**, **5**, and **9** exhibiting IC_50_ values in the low micromolar range. Enzymatic assays revealed that compounds **4** and **9** showed strong VEGFR-2 inhibition (97.9% and 96.4%, respectively), indicating apoptosis-inducing effects. **Conclusions:** The synthesized indolyl-based hybrid heterocycles represent a promising chemotype with in vitro cytotoxic activity and VEGFR-2 inhibitory effects, supporting further investigation, optimization, and mechanistic studies to evaluate their potential lead for anticancer drug development.

## 1. Introduction

Cancer is a vast category of dreadful illnesses characterized by uncontrolled cell growth and the potential to invade or spread to other tissues [1,2]. It is a leading cause of mortality worldwide [3,4,5,6]. Despite advances in chemotherapy, radiation, targeted therapy, and surgery, treatment outcomes can be limited by suboptimal efficacy and adverse effects [7,8]. Currently, some of these techniques, such as targeted therapy, have frequently poor efficacy and unfavorable side effects [9].

Breast cancer is one of the most common cancers worldwide and can affect women at any age, with risk increasing as life expectancy rises. Over the past five years, nearly 7.8 million women have been diagnosed with breast cancer. It was characterized by a high degree of variation in the likelihood of getting the illness [10], physiological characteristics, clinical behavior, and histological morphology. Furthermore, chemotherapy can have a significant negative impact on many cancer patients. Consequently, extensive research over the past decade has investigated breast cancer at the cellular, tissue, molecular, and clinical levels, making it one of the most widely studied diseases in oncology. Despite substantial advances, tumor remains poorly understood and unclear neoplasms. The goal is to reduce the worldwide breast cancer mortality rate by preventing 25% of deaths among women under the age of 70 by 2030 and 40% by 2040. Comprehensive breast cancer management, prompt diagnosis, and health promotion for early detection are the three principles that support achievement of this goal [11]. Therefore, it is critical to design and synthesize novel anti-cancer drugs with minimal side effects to enhance treatment efficiency and improve patient outcomes [12].

Breast cancer cells were known to proliferate when exposed to vascular endothelial growth factor (VEGF). The structure of VEGFR-1, VEGFR-2, and VEGFR-3 receptors is structurally and functionally similar to platelet-derived growth factor receptors (PDGFRs) [13]. They are therefore important angiogenesis intermediates [14,15]. One arsenal of kinases that is widely known to be connected to the onset and spread of breast cancer is VEGFR-2 kinase [16]. Since VEGFR-2 is essential for apoptosis and is significantly up-regulated in several solid tumor types, inhibiting VEGFR-2 has become a popular strategy for finding novel treatments for a variety of apoptosis-dependent cancers [17].

Heterocyclic compounds that contain nitrogen atoms have become more well-known in the medicinal and synthetic industry sectors, due to their various biological activities [18]. For instance, these activities consist of anti-tubercular activities [19], anti-HIV [20,21], anti-diabetic [22], anticancer [23,24], anti-microbial [25,26], and anti-malarial [27]. The indole scaffolds are one of the nitrogen-containing heterocycles with significant anticancer activity [28], anti-inflammatory [29], and anti-tuberculosis [30]. Numerous natural compounds, like vinblastine and vincristine, include the interesting indole moiety, which is recognized for its anticancer properties and can be utilized to treat cancers of the testicles, breast, ovaries, and head and neck [31,32,33].

Recent research has focused on indole derivatives with promising anti-breast cancer properties. Current research focuses specifically on BC, providing an explanation of the various ways that different indole derivatives work, including aromatase inhibitors, tubulin inhibitors, targeting estrogen receptors, microtubule inhibitors, apoptosis induction, DNA-binding mechanisms, PI3K/AkT/NFkB/mTOR inhibition, and inhibitors of HDAC [34].

**Figure 1:**
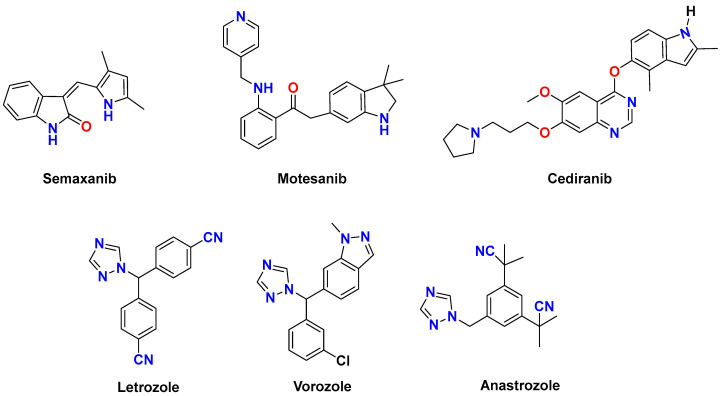
Typical pharmaceutical products with indole and 1,2,4-triazole components for the treatment of breast cancer.

The FDA has approved several indoles and triazole derivatives such as VEGFR-2 tyrosine kinase inhibitors, including Semaxanib, Indibulin, Motesanib, Panobinostat, Cediranib, Letrozole, Vorozole, and Anastrozole [35,36]. Tyrosine kinase inhibitors like Semaxanib are used to treat cancer [37,38,39,40]. Motesanib is a vascular inhibitor that specifically targets VEGFR-1,2,3 and acts as an adversary of c-KIT and PDGFR to directly prevent tumor growth [41]. Additionally, Cediranib is a strong tyrosine kinase inhibitor of VEGF receptor [42,43,44]. Letrozole, Vorozole, and Anastrozole have triazole moieties (Figure 1). The FDA has authorized these inhibitors for the endocrine therapy of postmenopausal women with breast cancer in both its early and advanced stages [45,46]. Triazole’s nitrogen atom is a critical element through its interactions with the iron of the aromatase’s heme.

**Figure 2:**
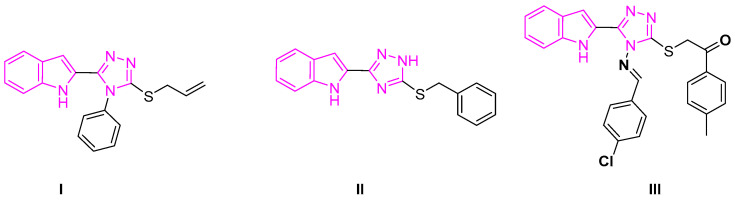
The active alkylated indole-triazole derivatives.

The alkylated indole-triazole system demonstrated strong anti-cancer activities (Figure 2). For instance, 3-(allylsulfanyl)-4-phenyl-5-(1*H*-indol-2-yl)-1,2,4-triazole **I** and its derivatives showed promising anti-breast cancer and antiproliferative effects [47]. The anti-proliferative effect of 3-benzylsulfenyl-5-(1*H*-indol-2-yl)–2H-1,2,4-triazole **II** against cancer cell lines of MCF-7 and HEPG-2 was encouraging [48]. Indolyl-triazole **III** and its derivatives showed a strong inhibition of VEGFR-2, which may have anti-renal cancer properties [49].

Building on our ongoing research into novel indole-based anticancer agents, we designed and synthesized a series of new S-alkylated derivatives derived from indole-1,2,4-triazoles. We assumed that the incorporation of S-alkyl groups, which enhance the lipophilicity and cellular permeability of these molecules, would thereby improve their anticancer efficacy. The synthesis strategy involved regioselective S-alkylation of the indole-1,2,4-triazole scaffold under the reaction conditions, which allowed a diverse range of alkyl substituents to be introduced. Structural characterization using ^1^H and ^13^C NMR, mass spectrometry, and elemental analysis unequivocally confirmed the successful formation of the target derivatives. The spectroscopic data were fully consistent with the proposed structures, and the elemental analyses were reasonable with the calculated values, thereby verifying both the identity and purity of the synthesized compounds.

Subsequent biological evaluations were then performed against breast cancer cell lines, revealing that several S-alkylated compounds exhibited significant cytotoxicity and induced cell cycle arrest. Notably, the promising derivatives inhibited cell proliferation as well as activated apoptotic pathways, as evidenced by increased caspase activity and modulation of the important key apoptotic markers. Our preliminary results highlight the potential of novel indolyl-1,2,4-triazole-based S-alkylated compounds as effective anticancer agents and warrant further investigation into their structure–activity relationships and in vivo efficacy.

## 2. Results and Discussion

### 2.1. Synthesis

**Scheme 1:**
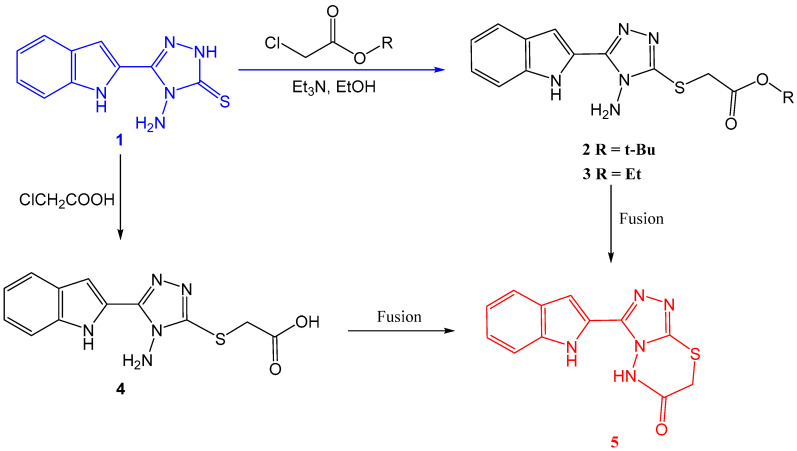
Alkylation of 4-amino-5-indolyl)-1,2,4-triazole-3-thione **1**.

4-Amino-5-(1*H*-indol-2-yl)-2,4-dihydro-3*H*-1,2,4-triazole-3-thione **1** was alkylated by tert-butyl bromoacetate or ethyl chloroacetate in the presence of Et_3_N in ethanol, affording the S-alkylated product **3** [50]. Chloroacetic acid reacted with **1** in the presence of potassium carbonate, affording the acid **4**. Fusion of esters **2** and **3**, or of the acid **4,** for three minutes, afforded **5** in excellent yield (Scheme 1). The structural confirmation of compounds **2**, **3**, and **5** agreed with the reported data [51]. The NMR of **4** displayed the Thio methylene protons (SCH_2_) at 4.04 ppm and the respective carbon at 33.45 ppm. Protons of NH_2_ appeared at 6.28 ppm. Protons of the indole NH and carboxylic group (COOH) showed at 11.72 and 12.87 ppm. The carbonyl carbon of the acid appeared at 169.8 ppm.

The single-crystal analysis of compounds **2**, **3**, and **5** was reported explicitly in our recent publications [51,52]. The crystal data showed that compounds **2** and **3** crystallized as a triclinic crystal system with primitive P 1 space group, while compound **5** was crystallized in the orthorhombic crystal system and Pbca space group. The unit cell parameters of crystals 2 and 3, respectively, were *a* = 5.43580(10) Å and 5.9924(2) Å, *b* = 10.4214(3) Å and 8.7769(3) Å, and *c* = 14.8371(3) Å and 29.2084(11) Å. Whereas, parameters of the unit cell **5** are *a* = 13.6036(3) Å, *b* = 7.8245(3) Å, and *c* = 21.3279(8) Å.

**Scheme 2:**
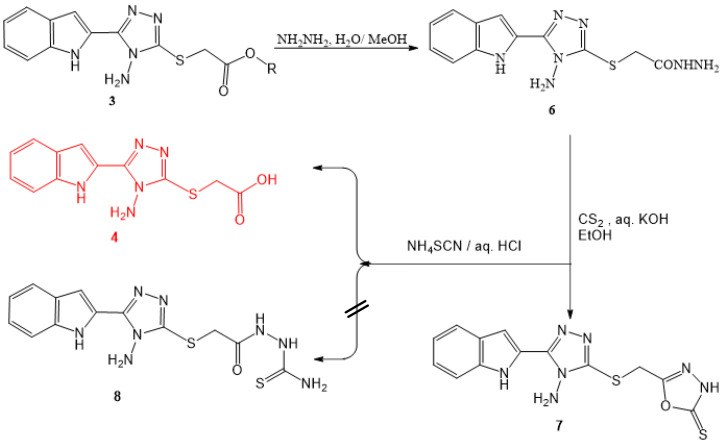
Synthesis of 5-(4-Amino-5-(indol-2-yl)-1,2,4-triazol-3-ylthiomethyl)-1,3,4-oxadiazol-2(3H)-thione **7**.

Hydrazide **6** is formed by the reaction of the esters **2** or **3** with NH_2_NH_2_·H_2_O in ethanol. Refluxing hydrazide **6** with ammonium thiocyanate in aqueous hydrochloric acid does not afford the thiosemicarbazide **8**; it gives the acid **4.** Refluxing the hydrazide **6** with CS_2_ in ethanol contains aqueous potassium hydroxide furnish 5-(4-Amino-5-(1H-indol-2-yl)-1,2,4-triazol-3-ylthiomethyl)-1,3,4-oxadiazol-2(3H)-thione **7** (Scheme 2). The NMR of **6** displayed the hydrazino-group (–NHNH_2_) at 4.32 and 9.34 ppm, the NH_2_ group at 6.28 ppm, and the indole NH protons at 11.74 ppm. A carbon of a carbonyl group appeared at 166.5 ppm [52]. NMR of compound **7** displayed a new proton signal at 14.49 ppm for NH of the new triazole ring. The thiocarbonyl carbon (C=S) of the formed triazoles-thione ring was found at 177.8 ppm.

**Scheme 3:**
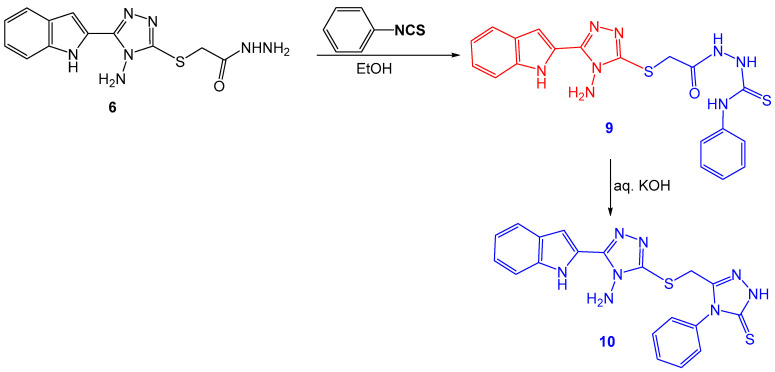
Synthesis of 5-(((4-amino-5-(indol-2-yl)-triazol-3-yl)thio)methyl)-4-phenyl-2,4-dihydro-1,2,4-triazole-3-thione **10**.

Reaction of phenyl isothiocyanate with hydrazide **6** afforded the thiosemicarbazide **9**, which was cyclized to form compound **10**, which contains indole and two 1,2,4-triazole rings in one aromatic system (Scheme 3). The thiosemicarbazide **9** NMR displayed the thiosemicarbazide proton signals (3 NH) at 9.34 and 10.37 ppm. The carbonyl carbon (C=O) and thiocarbonyl carbon (C=S) are found at 167.3 and 180.7 ppm. The NMR of the indolyl-triazolyl-triazole-thione **10** revealed the thiomethylene protons at 4.28 ppm, the protons of the amino group (NH_2_) at 6.23 ppm, and a new NH signal appeared at 13.85 ppm for the formed triazole-thione ring. The thiocarbonyl carbon (C=S) showed at 168.2 ppm.

### 2.2. Anticancer Activity Simulation Analysis

#### 2.2.1. Docking Analysis

**Figure 3:**
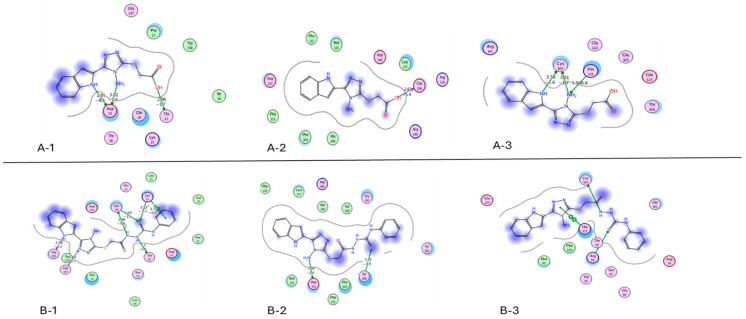
2D docking interaction diagrams of compounds **4** ((**A-1**): Bax, (**A-2**): Bcl-2, (**A-3**): Caspase-3) and compound **9** ((**B-1**): Bax, (**B-2**): Bcl-2, (**B-3**): Caspase-3), showing key hydrogen bonds and interacting amino acid residues in each binding site.

A molecular docking exploration was performed to investigate the purpose of investigating the binding disposition of synthetic chemicals. The docking results show that compounds **4** and **9** have strong binding towards the binding sites of Bax (PDB = 6EB6), Bcl2 (PDB = 6O0K), and Caspase-3 (PDB = 6BDV). The docking interaction of hit compounds **4** and **9** with Bax, Bcl2, and Caspase-3. Inside these figures, **A** represents the 2D interaction (Figure 3), **B** represents the 3D interaction, **C** represents the compound inside the protein pocket, and **D** represents the 3D compound on the surface of the protein. (Supplementary Figures S24–S29) Interestingly, compounds **4** and **9** form good interactive binding modes with the essential amino acids. The acid **4** interacts with Bax through three hydrogen bonds, two with Asp 53 and one with Thr 22 (bond lengths 2.95, 3.02, and 2.96 Å). The interaction of **4** with Bcl2 through hydrogen bonding with GLU 136 (bond length 2.83 Å). While the interaction of **4** with Caspase-3 is associated with two hydrogen bonds with CYS 163 and one hydrogen bond with HIS 121 (bond lengths 3.56, 3.51, and 3.04 Å). Hit compound **9** interacts with Bax through six hydrogen bonds with GLY 156, GLN 18, THR 56, GLY 157, GLN 18, LYS 21, and LYS 21 (bond lengths: 2.98, 2.89, 3.00, 3.64, 3.69, 3.53, and 3.71 Å, respectively). In this regard, compound **9** displays its interaction with Bcl2, the two hydrogen bonds with ASP 111 and TYR 108 (bond lengths: 3.09 and 3.79 Å). Moreover, compound **9** revealed the interaction with Caspase-3 through four hydrogen bonds with CYS 163, ARG 64, ARG 64, and HIS 121 (bond lengths: 3.67, 3.58, 4.36, and 3.97). As a result, compounds **4**, **5**, and **9** were nominated for the in vitro anticancer activity investigations.

#### 2.2.2. Cytotoxicity Assay

Using the MTT test, the cytotoxic activity of the synthesized compounds was evaluated against MCF-7 and HepG2 cell lines, revealing significant dose-dependent inhibition of cancer cell viability. Among the tested derivatives, compounds **4**, **5**, and **9** displayed the highest cytotoxic effects against cells of HepG2 with IC_50_ values of 0.2715, 0.3167, and 0.4101 μM. Among the tested derivatives, compounds **1**, **3**, **5**, **9**, and **10** displayed the highest cytotoxic effects against MCF7 cells, with IC_50_ values of 0.2128, 0.3843, 0.2373, 0.4994, and 0.2506 μM. The potency of these compounds may be attributed to their enhanced lipophilicity and favorable electronic distribution, allowing for efficient cell membrane penetration and intracellular accumulation.

##### In Vitro Enzyme Assay (Inhibition%)

Measurement of VEGFR-2 kinase activity

Moreover, compounds **4** and **9** strongly inhibit VEGFR-2 by 97.9% and 96.4% at IC_50_ 0.59 and 1.23 μM, respectively (Table 1). While compounds **1**, **5**, and **10** have inhibition activity, ranging from 83.9 to 87.8%.

##### In Vitro Analysis by Flow Cytometry

**Figure 4:**
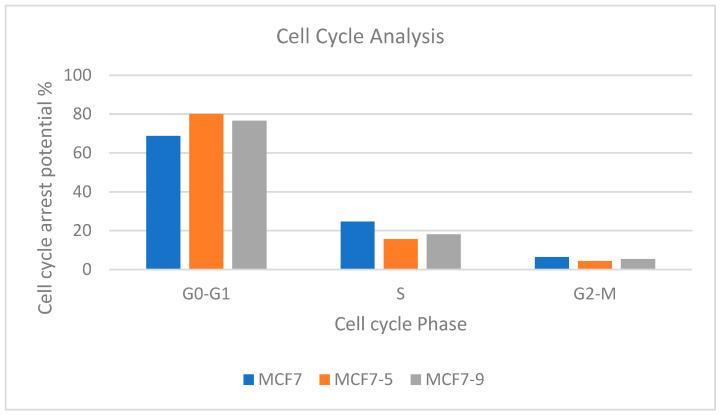
Cell-cycle distribution of MCF-7 cells after treatment with compounds **5** and **9**. Cells were treated for 24 h with compound **5** (0.24 µM) or compound **9** (0.49 µM) (MCF-7 IC_50_ concentrations), stained with propidium iodide (PI), and analyzed by flow cytometry. Quantitative phase distributions (G0/G1, S, and G2/M) are presented as mean ± SD (n = 3) and summarized in Table 2. Statistical significance versus untreated control was assessed by one-way ANOVA with Tukey’s multiple comparisons test (*p* < 0.05).

A series of events make up the cell cycle, which includes DNA replication, cell division, and ultimately the production of two daughter cells. To reduce the chance of generic medication side effects when treating cancer, it is attractive to build new anti-proliferative agents that can regulate the progression of the cell cycle and death. Consequently, the most effective anticancer substances (**5**) and (**9**), which showed notable activity on the cell line of MCF7, were also chosen for more research into the ability of these compounds to cause cell cycle arrest. Compounds **5** and **9** arrested the cell cycle at the S and G2/M phase, where the cell population levels of p53 wild-type cell lines MCF7 decreased from (24.74–6.48%) for the control to (15.68–4.33%) and (18.06–5.37%) for **5** and **9**, respectively. However, the two compounds under investigation showed an extra arrest in the G0–G1 phase of the cell cycle, which raised the two compounds’ cell population levels (79.97, 76.58%), respectively, compared to the control (68.79%) (Figure 4, Table 2).

**Figure 5:**
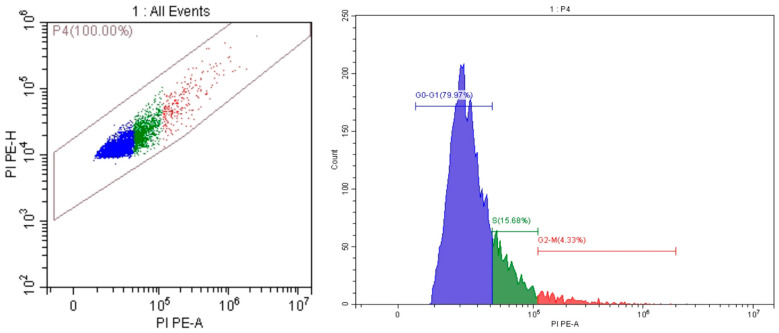
Representative PI-based cell-cycle histogram of MCF-7 cells treated with compound **5**. Cells were treated for 24 h with compound **5** (0.24 µM; MCF-7 IC_50_) and processed in parallel with untreated control cells. DNA content was assessed by PI staining and flow cytometry. The histogram shown is representative of three independent experiments (n = 3); the corresponding quantitative phase distributions and statistics are reported in Figure 4/Table 2 (one-way ANOVA with Tukey’s test).

**Figure 6:**
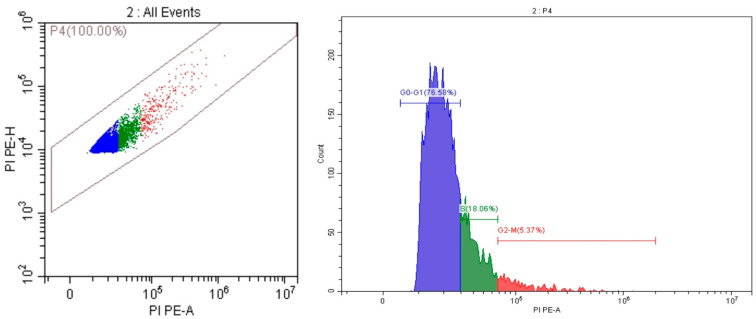
Representative PI-based cell-cycle histogram of MCF-7 cells treated with compound **9**. Cells were treated for 24 h with compound **9** (0.49 µM; MCF-7 IC_50_) and processed in parallel with untreated control cells. DNA content was assessed by PI staining and flow cytometry. The histogram shown is representative of three independent experiments (n = 3); the corresponding quantitative phase distributions and statistics are reported in Figure 4/Table 2 (one-way ANOVA with Tukey’s test).

Table 2 summarizes the distribution of untreated MCF-7 cells and cells treated with compounds **5** and **9** across the G0/G1, S, and G2/M phases. Untreated cells showed 68.79% in G0/G1, 24.74% in the S phase, and 6.48% in G2/M. Treatment with compound **5** increased the G0/G1 population to 79.97% and reduced the S phase and G2/M populations to 15.68% and 4.33%, respectively. Similarly, compound **9** increased the G0/G1 fraction to 76.58%, with S and G2/M fractions of 18.06% and 5.37%. These shifts indicate that both compounds induce cell-cycle arrest, particularly enhancing the accumulation of cells in the G0/G1 phase, a typical indication of antiproliferative activity and the onset of apoptotic processes (as shown in Figure 5 and Figure 6).

The shift towards increased G0/G1-phase accumulation, coupled with the reduction in S and G2/M populations, suggests that the compounds affect cell cycle progression, which may lead to apoptosis and a reduction in overall cell proliferation. This evidence reinforces the potential of these compounds as promising anticancer agents.

**Figure 7:**
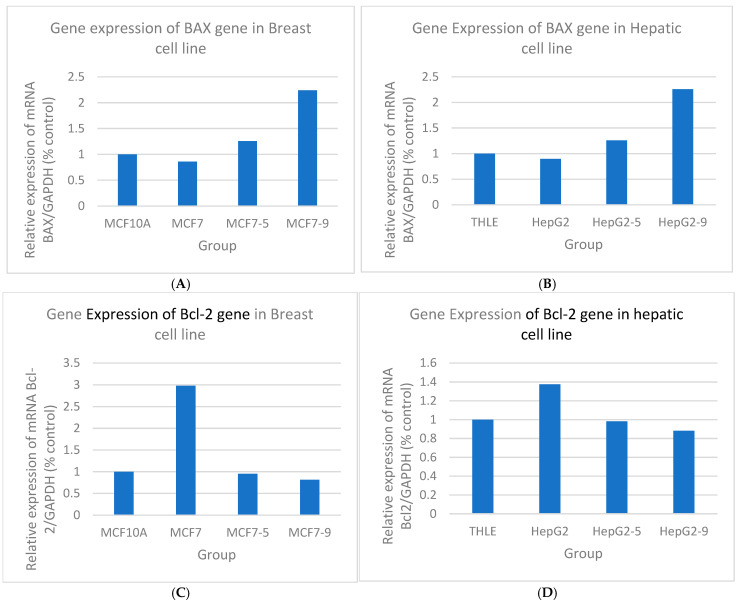
Relative mRNA expression of BAX and Bcl-2 in (**A**,**C**) MCF-7 and (**B**,**D**) HepG2 cells after treatment with compounds **5** and **9**. Expression levels were normalized to GAPDH and calculated using the 2^−ΔΔCt^ method. Data are mean ± SD (n = 3). Statistical significance was assessed by one-way ANOVA with Tukey’s multiple comparisons test; *p* < 0.0001 versus untreated cancer cells.

Gene expression analysis:

Figure 7 shows the relative mRNA expression of the apoptotic regulators BAX and Bcl-2 in MCF-7 and HepG2 cells after treatment with compounds **5** and **9**. Compared with untreated cancer cells, both compounds significantly upregulated BAX and downregulated Bcl-2 (**** *p* < 0.0001), shifting the balance toward apoptosis.

Further investigation using quantitative PCR (qPCR) corroborated these findings, demonstrating that not only do compounds **5** and **9** restore BAX expression, but compound **4** similarly enhances the expression of this pro-apoptotic gene, while all treatments lead to a significant decrease in Bcl-2 expression. These molecular alterations suggest that these compounds exert their anticancer effects by directly influencing the intrinsic (mitochondrial) pathway of apoptosis. It is postulated that the structural features of these compounds enable strong hydrogen-bonding interactions with apoptotic regulatory proteins, thereby facilitating conformational changes that trigger mitochondrial outer membrane permeabilization.

The resultant release of cytochrome c from the mitochondria would then activate downstream Caspases, culminating in programmed cell death. This mechanism of action is particularly critical in drug-resistant malignancies, where evasion of apoptosis is a common survival strategy. By restoring the balance between pro- and anti-apoptotic factors, compounds **5**, **9**, and **4** not only promote cell death but also potentially overcome the resistance mechanisms that allow cancer cells to evade conventional therapies.

Thus, the ability of these compounds to modulate apoptosis underscores their potential as effective anticancer agents. Their action in reestablishing apoptotic sensitivity in cancer cells could be pivotal in the development of new therapeutic strategies aimed at targeting resistant tumors, reinforcing the notion that the modulation of apoptotic pathways is a defining characteristic of promising anticancer compounds.

## 3. Materials and Methods

### 3.1. Materials

All reagents and solvents were purchased from Sigma-Aldrich (St. Louis, MO, USA) and/or Al-Nasr Co. (Giza, Egypt) and used without further purification unless otherwise stated. Reaction progress was monitored by thin-layer chromatography (TLC) on silica gel 60 F254 plates and visualized under UV light (254/365 nm). Melting points were determined on an SMP10 melting point apparatus and are uncorrected. ^1^H and ^13^C NMR spectra were recorded on Bruker spectrometers (frequencies as indicated) in DMSO-*d*_6_ or CDCl_3_; chemical shifts (δ) are reported in ppm relative to residual solvent signals. Elemental analyses (C, H, N) were performed using a Flash EA-1112 analyzer (Thermo Fisher Scientific, Rodano, Italy). Mass spectra (EI/HRMS) were recorded as indicated in the compound characterization. Unless otherwise noted, products were purified by filtration and/or recrystallization from DMF/ethanol.

### 3.2. Experimental

#### 3.2.1. Synthesis of Tert-Butyl 2-((4-Amino-5-(1*H*-indol-2-yl)-4*H*-1,2,4-triazol-3-yl)thio)acetate 2 and Ethyl 2-((4-Amino-5-(1*H*-indol-2-yl)-4*H*-1,2,4-triazol-3-yl)thio)acetate 3

Compounds **2** and **3** were synthesized as reported in our recent work [50]. Briefly, a mixture of 4-amino-5-(1*H*-indol-2-yl)-1,2,4-triazol-3(2*H*)-thione **1** (1.0 mmol) and triethylamine (1.2 mmol) in absolute ethanol was stirred at room temperature for 1 h. The appropriate halo ester (1.1 mmol) was then added, and the reaction mixture was stirred at room temperature overnight. After completion, ethanol was removed under vacuum using a rotary evaporator, and cold water (100 mL) was added to the residue. The resulting precipitate was collected by filtration, washed with water, and air-dried at room temperature. The crude product was purified by recrystallization from ethanol to afford compounds **2** and **3** as pure crystalline solids.

#### 3.2.2. Synthesis of 4-Amino-3-(carboxymethylsulfanyl)-5-(1*H*-indol-2-yl)-1,2,4-triazole 4

A mixture of compound **1** (1.0 mmol) and K_2_CO_3_ (1.2 mmol) in ethanol (10 mL) was stirred at room temperature for 1 h. Chloroacetic acid (1.2 mmol) was added, and the reaction mixture was refluxed for 3 h. After cooling to room temperature, the solvent was removed under vacuum using a rotary evaporator. Ice water (50 mL) was added to the residue, and the mixture was acidified with 10% (*v*/*v*) aqueous HCl until the pH reached 3. The resulting precipitate was collected by filtration, washed with water, and recrystallized from DMF/EtOH to give compound **4**. Yield: 78%, m.p. 240–241 °C. ^1^H NMR (DMSO-*d*_6_, 300 MHz) δ 4.04 (SCH_2_), 6.28 (s, 2 H, NH_2_, D_2_O exchangeable), 7.03 (dd, 1 H, *J* = 7.8, *J* = 7.5 Hz), 7.16 (dd, 1 H, *J* = 7.5, *J* = 8.1 Hz), 7.28 (d, 1 H, *J* = 1.5 Hz), 7.45 (d, 1 H, *J* = 8.1 Hz), 7.61 (d, 1 H, *J* = 7.8 Hz), 11.72 (br. s, 1 H, NH, D_2_O exchangeable), 12.87 (br. s, 1 H, COOH, D_2_O exchangeable); ^13^C NMR (DMSO-*d*_6_, 100 MHz) δ 33.5 (SCH_2_), 102.3, 111.9, 119.6, 120.7, 122.8, 123.9, 127.6, 136.5, 149.4, 152.9, 169.8 (C=O); HRMS (FAB + ve) calcd for C_12_H_12_N_5_O_2_S (M + 1): 290.0712 Found: 290.0690.

#### 3.2.3. Synthesis of the 3-(1*H*-Indol-2-yl)-7*H*-[1,2,4]triazolo [3,4-b][1,3,4]thiadiazin-6-ol 5

Previously [51], compound **5** was obtained in 81% yield from reaction of 4-amino-5-(1*H*-indol-2-yl)-1,2,4-triazol-3(2*H*)-thione **1** (1.0 mmol) and ethyl chloroacetate or tert-butyl bromoacetate (1.2 mmol) in ethanol (10 mL) in the presence of K_2_CO_3_ (1.2 mmol) as base (Scheme 1), and single-crystal X-ray diffraction analysis showed that it was present in the enol form in the solid state.

In the present work, the alkylated esters (**2** or **3**) or the acid (**4**) (1.0 mmol) were fused on a hotplate in open air at atmospheric pressure for 3 min. After cooling to room temperature, the crude product was purified by recrystallization from DMF/ethanol to afford compound **5** as crystals. Yield: >92%; m.p. 291–292 °C. Full ^1^H and ^13^C NMR data are provided in the Supplementary Materials.

#### 3.2.4. Synthesis of 2-((4-Amino-5-(1*H*-indol-2-yl)-4*H*-1,2,4-triazol-3-yl)thio)acetohydrazide 6

Compound **6** was synthesized according to our previously reported protocol [52], in which all characterization data and analytical details have been described. Briefly, ethyl 2-((4-amino-5-(1*H*-indol-2-yl)-4*H*-1,2,4-triazol-3-yl)thio)acetate **3** (2.0 mmol) was treated with hydrazine hydrate (3 mL) and refluxed for 1 h. After completion, excess solvent and unreacted hydrazine were removed under vacuum using a rotary evaporator, and the crude product was crystallized from ethanol to afford compound **6** as a pure solid. Yield: 87%; m.p. 248–250 °C. Full ^1^H and ^13^C NMR data are provided in the Supplementary Materials.

#### 3.2.5. 5-(4-Amino-5-(1*H*-indol-2-yl)-1,2,4-triazol-3-ylthiomethyl)-1,3,4-oxadiazol-2(3*H*)-thione 7

Hydrazide **6** (2.0 mmol), carbon disulfide (5.0 mmol) and aqueous KOH (4.0 mmol) were refluxed in ethanol (30 mL) for 4 h. After cooling, the reaction mixture was concentrated by evaporation to approximately half of its original volume Ice water (50 mL) was added, and the mixture was acidified with 10% (*v*/*v*) aqueous HCl until pH equals 3. The precipitate was collected by filtration and recrystallized from DMF/EtOH to form and purify compound **7**. Yield: 66%, m.p. 218–220 °C. ^1^H NMR (DMSO-*d*_6_, 300 MHz) δ 4.55 (s, 2 H, SCH_2_), 6.20 (br., 2 H, NH_2_), 7.04 (dd, 1 H, *J* = 7.8, *J* = 7.2 Hz), 7.17 (dd, 1 H, *J* = 7.2, *J* = 8.1 Hz), 7.31 (s, 1 H), 7.46 (d, 1 H, *J* = 8.1 Hz), 7.62 (d, 1 H, *J* = 7.8 Hz), 11.78 (br.s, 1 H, NH), 14.49 (br.s, H, NH); ^13^C NMR (DMSO-*d*_6_, 100 MHz) δ 25.1 (SCH_2_), 102.8, 111.9, 119.7, 120.9, 123.0 123.5, 127.1, 136.6, 149.7, 151.2, 160.7, 177.9 (C=S); HRMS (EI) calcd for C_13_H_10_N_7_OS_2_ (M^+^): 344.0388 Found: 344.0395.

#### 3.2.6. Synthesis of 1-(4-Amino-5-(1*H*-indol-2-yl)-1,2,4-triazol-3-ylthioacety1)-4-phenylthiosemicarbazide 9

Hydrazide **6** (2.0 mmol) was refluxed with phenyl isothiocyanate (2.0 mmol) in ethanol (10 mL) for 3 h. After cooling to room temperature, the precipitate was collected by filtration and recrystallized from DMF/EtOH to afford compound **9**. Yield: 92%, m.p. 227–228 °C. ^1^H NMR (DMSO-*d*_6_, 300 MHz) δ 3.87 (s, 2 H, SCH_2_), 6.28 (s, 2 H, NH_2_), 7.01–7.60 (m, 10 H), 9.34 (2br.s, 2 H, NHNH), 10.37 (br. s, 1 H, NH), 11.67 (br.s, 1 H, NH); ^13^C NMR (DMSO-*d*_6_, 100 MHz) δ 34.1 (SCH_2_), 102.6, 111.9, 119.7, 120.8, 122.9, 123.8, 125.1, 125.6, 127.5, 128.0, 136.5, 138.9, 149.3, 153.0, 167.4 (C=O), 180.72 (C=S); HRMS (FAB -ve) calcd for C_19_H_17_N_8_S_2_O (M-1): 437.0967 Found: 437.0953.

#### 3.2.7. Synthesis of 4-Amino-5-(1*H*-indol-2-yl)-3-(4-phenyl-3-thioxo-1,2,4-triazol-5-ylmethylsulfanyl)-1,2,4-triazole 10

A mixture of thiosemicarbazide **9** (2.0 mmol) in aqueous KOH (4.0 N, 50 mL) was refluxed for 4 h, then allowed to cool to room temperature. The mixture was acidified with HCl (10%) until pH 3, and the formed precipitate was collected by filtration and recrystallized from DMF/EtOH to form compound **10**. Yield: 71%, m.p. 253–255 °C. ^1^H NMR (DMSO-*d*_6_, 300 MHz) δ 4.28 (s, 2 H, SCH_2_), 6.23 (s, 2 H, NH_2_), 7.03 (dd, 1 H, *J* = 7.8, *J* = 7.2 Hz), 7.17 (dd, 1 H, *J* = 7.2, *J* = 8.1 Hz), 7.28 (s, 1 H), 7.45 (d, 1 H, *J* = 8.1 Hz), 7.50–7.57 (m, 5 H), 7.61 (d, 1 H, *J* = 7.8 Hz), 11.75 (br.s, 1 H, NH), 13.85 (br. s, 1 H, NH); ^13^C NMR (DMSO-*d*_6_, 75 MHz) δ 26.0 (SCH_2_), 102.5, 111.9, 119.7, 120.8, 122.9, 123.8, 127.6, 128.4, 129.4, 129.6, 133.3, 136.5, 148.7, 149.5, 151.2, 168.2 (C=S); HRMS (FAB -ve) calcd for C_19_H_15_N_8_S_2_ (M^+^): 419.0861 Found: 419.0899.

### 3.3. Anticancer Activity

#### 3.3.1. Reagents and Chemicals

RPMI-1640 medium, fetal bovine serum (FBS), trypsin, and phosphate-buffered saline (PBS) were obtained from ICN Biomedicals Inc. (Aurora, OH, USA). Penicillin/streptomycin was purchased from Invitrogen (San Diego, CA, USA). The MTT reagent (CAS 298-93-1) was obtained from Yunbang Pharma (Changsha, Hunan, China). Doxorubicin was purchased from EBEWE Pharma (Unterach, Austria). Dimethyl sulfoxide (DMSO) was obtained from Merck (Darmstadt, Germany).

#### 3.3.2. Cell Culture

Human HepG2 (liver cancer) and MCF-7 (breast cancer) cell lines were obtained from the Al-Azhar Virology Research Center (Faculty of Medicine, Cairo, Egypt). Cells were maintained in RPMI-1640 supplemented with 10% FBS and penicillin/streptomycin (100 U/mL and 100 µg/mL, respectively) and cultured as adherent monolayers at 37 °C in a humidified atmosphere containing 5% CO_2_.

#### 3.3.3. Cell Viability (MTT) Assay

Cell viability was evaluated using the MTT reduction assay [53]. HepG2 and MCF-7 cells were seeded in 96-well plates (8000–10,000 cells/well; 100 µL complete medium) and allowed to attach overnight. The medium was then replaced with serum-free medium containing serial dilutions of the test compounds (0.195–25 µM; 1:2 dilution series). Stock solutions were prepared in DMSO, and the final DMSO concentration in all wells was kept below 0.5% (*v*/*v*). Control wells received vehicle only, and blank wells contained medium without cells for background subtraction.

After 24 h treatment, the medium was removed, and MTT reagent was added (0.5 mg/mL final concentration), followed by incubation for 4 h at 37 °C. Formazan crystals were dissolved in DMSO (150 µL/well), and absorbance was measured at 570 nm (reference 655 nm) using a STAT-Fax 2100 microplate reader (Awareness Technology, Inc., Palm City, FL, USA). Percentage cell viability was calculated relative to vehicle-treated controls. IC_50_ values were determined by nonlinear regression using GraphPad Prism (version 9). All experiments were performed in triplicate (n = 3), and data are reported as mean ± SD.

##### Assessment of In Vitro VEGFR-2 Inhibitory Activity

VEGFR-2 kinase inhibitory activity was assessed using a commercial VEGFR-2 kinase assay kit (Bioscience) according to the manufacturer’s instructions. Briefly, recombinant VEGFR-2 was incubated with the supplied substrate/ATP mixture in the presence of serial dilutions of the test compounds. Enzymatic control wells contained enzyme with inhibitor buffer only (no inhibitor), and blank wells contained all reagents except enzyme. Residual ATP was quantified using Kinase-Glo^®^ MAX (Promega, Madison, WI, USA), and luminescence was recorded using a microplate reader. Percentage inhibition was calculated relative to the enzymatic control, and IC_50_ values were obtained from concentration–response curves (n = 3). Sunitinib was included as a reference inhibitor.

##### In Vitro Cell Cycle Analysis by Flow Cytometry

Cell cycle distribution was analyzed in MCF-7 cells after treatment with compounds **5** and **9**. Following 24 h exposure, approximately 1 × 10^6^ cells were harvested by trypsinization, washed twice with ice-cold PBS (pH 7.4), and fixed in 60% ice-cold ethanol at 4 °C for 1 h. Fixed cells were washed with PBS and resuspended in PBS containing RNase A (50 µg/mL) and propidium iodide (PI; 10 µg/mL). After incubation in the dark for 30 min at 15–25 °C, samples were analyzed using a CytoFLEX™ flow cytometer (Beckman Coulter, Inc., Brea, CA, USA) with excitation/emission at 535/617 nm (FL2 channel). A minimum of 20,000 events was collected per sample, and cell-cycle distribution was calculated using Cytexpert™ v2.4.0.28 software.

##### Real-Time Quantitative PCR (RT-qPCR) Analysis

Total RNA was extracted from untreated and treated MCF-7 and HepG2 cells using QIAzol Lysis Reagent (QIAGEN, Hilden, Germany) according to the manufacturer’s protocol, and RNA concentration/purity were assessed using a DENOvix UV spectrophotometer (DeNovix Inc., Wilmington, DE, USA). First-strand cDNA was synthesized from equal amounts of RNA using the HiSenScript™ RH (-) cDNA Synthesis Kit (iNtRON Biotechnology, Seongnam, Republic of Korea). Quantitative real-time PCR (RT-qPCR) was performed on a CFX96 Deep Well Real-Time PCR System Bio-Rad Laboratories, Hercules, CA, USA). Primer sequences for BAX, Bcl-2 and GAPDH are listed in Table 3. Relative gene expression was calculated using the 2^−ΔΔCt^ method and normalized to GAPDH. Each condition was analyzed in triplicate (n = 3).

#### 3.3.4. Statistical Analysis

All quantitative data are presented as mean ± SD of three replicate measurements (n = 3) unless otherwise stated. IC_50_ values were obtained by nonlinear regression (four-parameter logistic model) using GraphPad Prism (version 9). For multi-group comparisons (e.g., RT-qPCR), statistical significance was assessed using one-way ANOVA followed by Tukey’s multiple comparisons test. A *p*-value < 0.05 was considered statistically significant.

## 4. Conclusions

A series of novel heterocyclic compounds incorporating indole, 1,2,4-triazole, thiadiazine, and 1,3,4-oxadiazole motifs was synthesized and evaluated for in vitro anticancer activity against MCF-7 and HepG2 cells. Several compounds showed low-micromolar cytotoxicity, induced cell-cycle perturbation, and modulated apoptotic markers (BAX upregulation and Bcl-2 downregulation). In addition, selected derivatives inhibited VEGFR-2, supporting a dual apoptosis/antiangiogenic profile. These findings warrant further optimization and mechanistic studies to assess their potential as anticancer leads. Structural analysis suggests that the unique arrangement of heterocyclic moieties facilitates strong hydrogen-bonding interactions with key regulatory proteins, thereby stabilizing conformational changes that favor apoptosis and impede survival signaling pathways. Such interactions likely contribute to the compounds’ ability to target the mitochondrial pathway, leading to the release of cytochrome c and subsequent activation of caspase cascades. The multifaceted mechanism of action of these heterocyclic scaffolds underscores their potential as next-generation anticancer agents. Their ability to simultaneously trigger intrinsic apoptotic pathways, modulate gene expression, and inhibit angiogenic processes presents a compelling therapeutic strategy for overcoming the limitations of current treatments, particularly in cases of drug-resistant cancers. These findings provide a strong rationale for further preclinical studies and optimization to translate these compounds into clinically viable therapies that could significantly improve outcomes for patients battling aggressive, resistant tumors.

## Figures and Tables

**Table 1:** Cytotoxic activity of the synthesized compounds against HepG2 and MCF-7 cell lines and inhibitory activity against VEGFR-2. Values are reported as mean ± SD (n = 3). Individual replicate IC_50_ values are provided in the Supporting Information (Tables S1–S3).

Compound	(HepG2) (IC_50_ µM)	(MCF-7) (IC_50_ µM)	VEGFR-2 (IC_50_ µM)	VEGFR-2 Inhibition (%)
**1**	1.8 ± 0.0028	0.21 ± 0.00027	2.3 ± 0.012	85.8
**2**	0.96 ± 0.0017	0.95 ± 0.0027		
**3**	2.1 ± 0.0065	0.35 ± 0.0034		
**4**	0.27 ± 0.002	0.97 ± 0.0023	0.59 ± 0.004	97.9
**5**	0.32 ± 0.0014	0.24 ± 0.00054	2.4 ± 0.014	83.9
**6**	1.5 ± 0.031	0.82 ± 0.00033		
**7**	5.8 ± 0.074	4.6 ± 0.0062		
**9**	0.41 ± 0.00048	0.49 ± 0.0019	1.2 ± 0.025	96.4
**10**	1.2 ± 0.0038	0.25 ± 0.00038	2.3 ± 0.017	87.8
Doxo.	0.16 ± 0.000033	0.1 ± 0.00066		
Sunitinib			0.32 ± 0.006	98.9

**Table 2:** MCF7 = untreated/vehicle control cells; MCF7-5 = MCF-7 cells treated with compound **5**; MCF7-9 = MCF-7 cells treated with compound **9**. Cells were treated for 24 h and stained with PI prior to flow-cytometric analysis. Data are presented as mean ± SD (n = 3 independent experiments). Statistical analysis was performed using one-way ANOVA followed by Tukey’s multiple comparisons test (*p* < 0.05).

Group/Phase	G0-G1	S	G2-M
MCF7	68.79 ± 0.34	24.74 ± 0.40	6.48 ± 0.16
MCF7-5	79.97 ± 0.51	15.68 ± 0.46	4.33 ± 0.24
MCF7-9	76.58 ± 0.46	18.06 ± 0.33	5.37 ± 0.30

**Table 3:** Primer Sequences for RT-qPCR.

Gene	Sequence	Tm (°C)	Product Size (bp)	Accession Number
*BAX*	F: 5′-GATTACAGACCCCAGGCAGG-3′ R: 5′-TGGCTCAAGTAGGACGGGTA-3′	54	130	NM_001291428
*Bcl-2*	F: 5′-GCAATGGGCACGAGTTTGTT-3′ R: 5′-AGTGTGTTCACCAGGCCAAA-3′	60	170	NM_000633
*GAPDH*	F: 5′-CCATCAACGACCCCTTCATT-3′ R: 5′-CACGACATACTCAGCACCAGC-3′	58	193	NM_001256799

## Data Availability

The original contributions presented in this study are included in the article/Supplementary Material. Further inquiries can be directed to the corresponding author.

## Supplementary Materials

The following supporting information can be downloaded at: https://www.mdpi.com/article/10.3390/ph19030418/s1, Figures S1–S6: ^1^H NMR, ^13^C NMR, DEPT, COSY, HMQC, and EIMS of compound **4**; Figures S7 and S8: ^1^H NMR and ^13^C NMR of compound **5**; Figures S9–S11: ^1^H NMR, ^13^C NMR, and DEPT135 NMR of compound **6**; Figures S12–S15: ^1^H NMR, ^13^C NMR, DEPT135 NMR, and HMQC NMR of compound **7**; Figures S16–S19: ^1^H NMR, ^13^C NMR, DEPT, and HMQC of compound **9**; Figures S20–S23: ^1^H NMR, ^13^C NMR, DEPT, and HMQC of compound **10**; Figures S24–S29: docking interactions of compound **4** with Bax, Bcl2, and Caspase-3 and of compound **9** with Bax, Bcl2, and Caspase-3 (interaction views include: B = 3D interaction, C = compound inside the protein pocket, D = compound on the protein surface); Figures S30–S32: HepG2 IC_50_, MCF-7 IC_50_, and VEGFR-2 IC_50_ plots; Figures S33–S35: representative SDS-PAGE gel used prior to immunoblotting and uncropped western blots for BAX (20 kDa) and Bcl-2 (26 kDa) expression in control breast cells, untreated MCF-7 cells, and MCF-7 cells treated with compounds **5** and **9**; Tables S1–S3: HepG2, MCF-7, and VEGFR-2 IC_50_ values (triplicate measurements) with calculated mean ± SD (n = 3).
