# Supplementary material for: Exploration of Novel Indole Compounds with Potential Activity Against Breast Cancer: Synthesis, Characterization and Anti-Cancer Activity Evaluation
Source: Pharmaceuticals (Basel). 2026 Mar 4;19(3):418. doi: 10.3390/ph19030418 (PMC13029243; doi:10.3390/ph19030418)
Supplement: Supplementary file 1 [file pharmaceuticals-19-00418-s001.zip › pharmaceuticals-4018387-supplementary.pdf]

Exploration of Novel Indole Compounds with Potential Activity Against Breast Cancer: Synthesis, Characterization and Anti-cancer Activity Evaluation

Eid E. Salama [1*], Ashtar A. Alrayes[1], Saad Alrashdi [1], Ahmed T.A. Boraei [2], Nagwa I. Ahmed [3], Salah Eid[1], Karam S. El-Nasser [1], Haitham Kalil [5*], and Ahmed A. M. Sarhan [4]

Spectroscopy and Structure Analysis

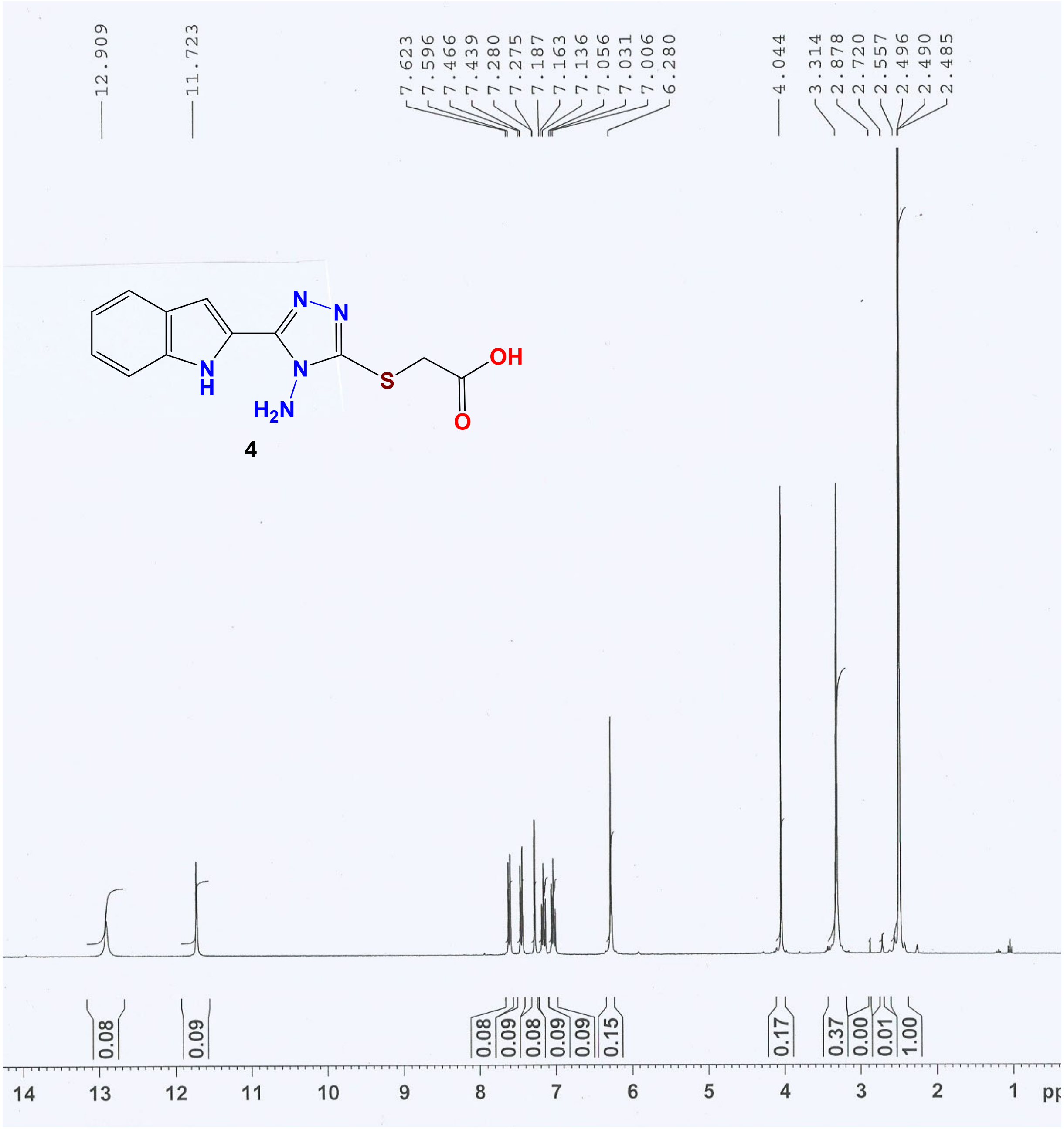

Figure S1. ^{1}H NMR of **4**

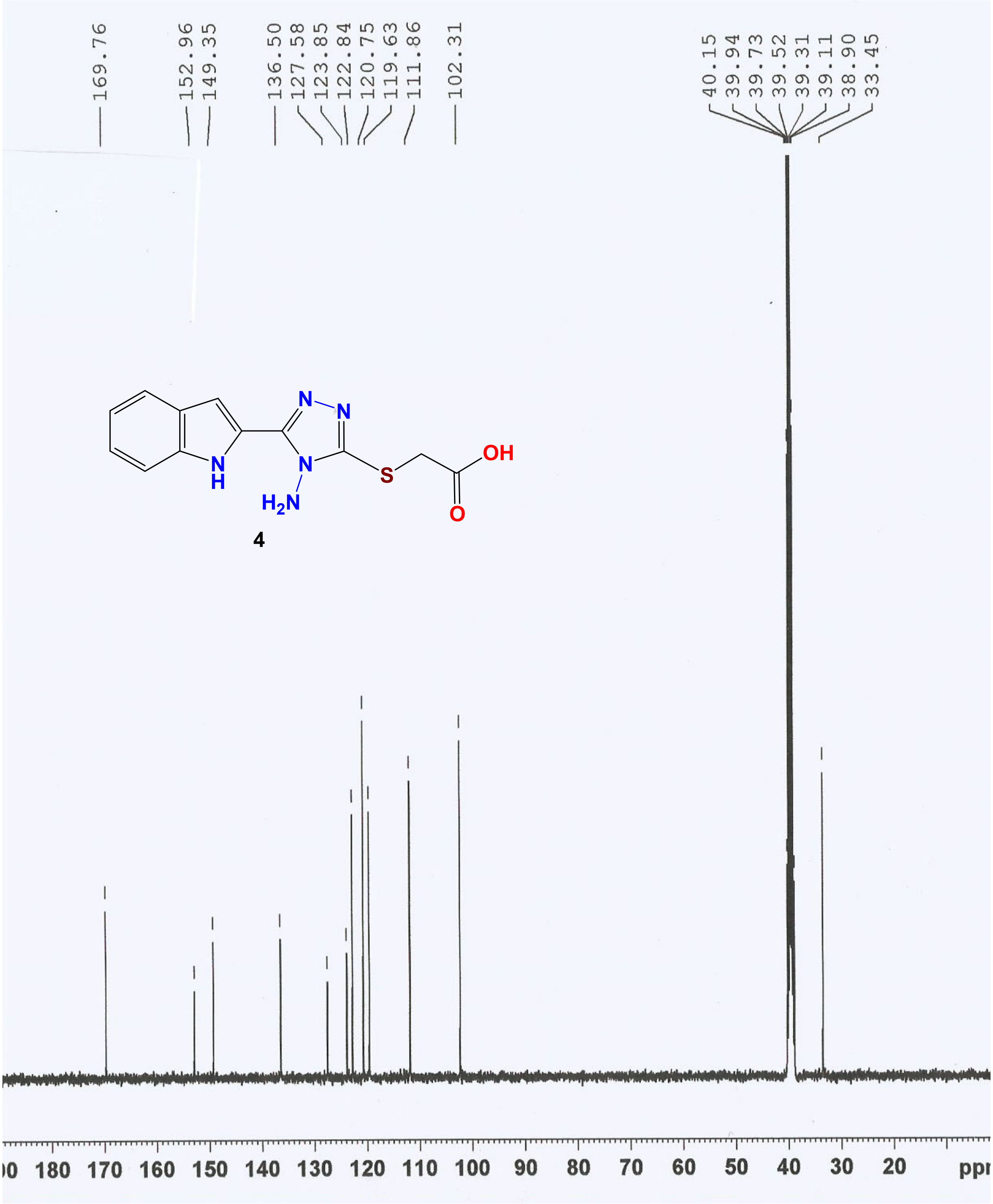

Figure S2. ^{13}C NMR of **4**

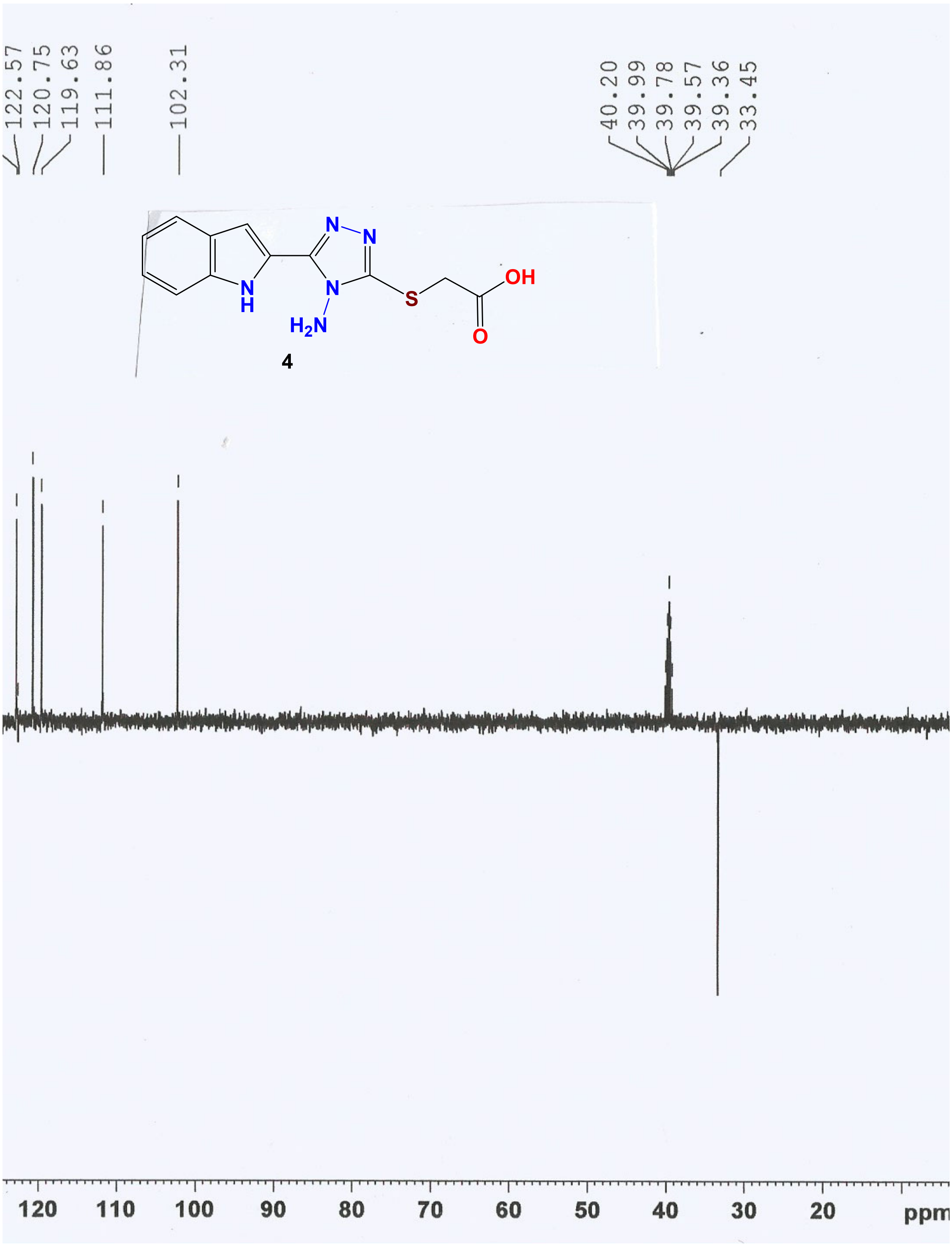

Figure S3. DEPT of **4**

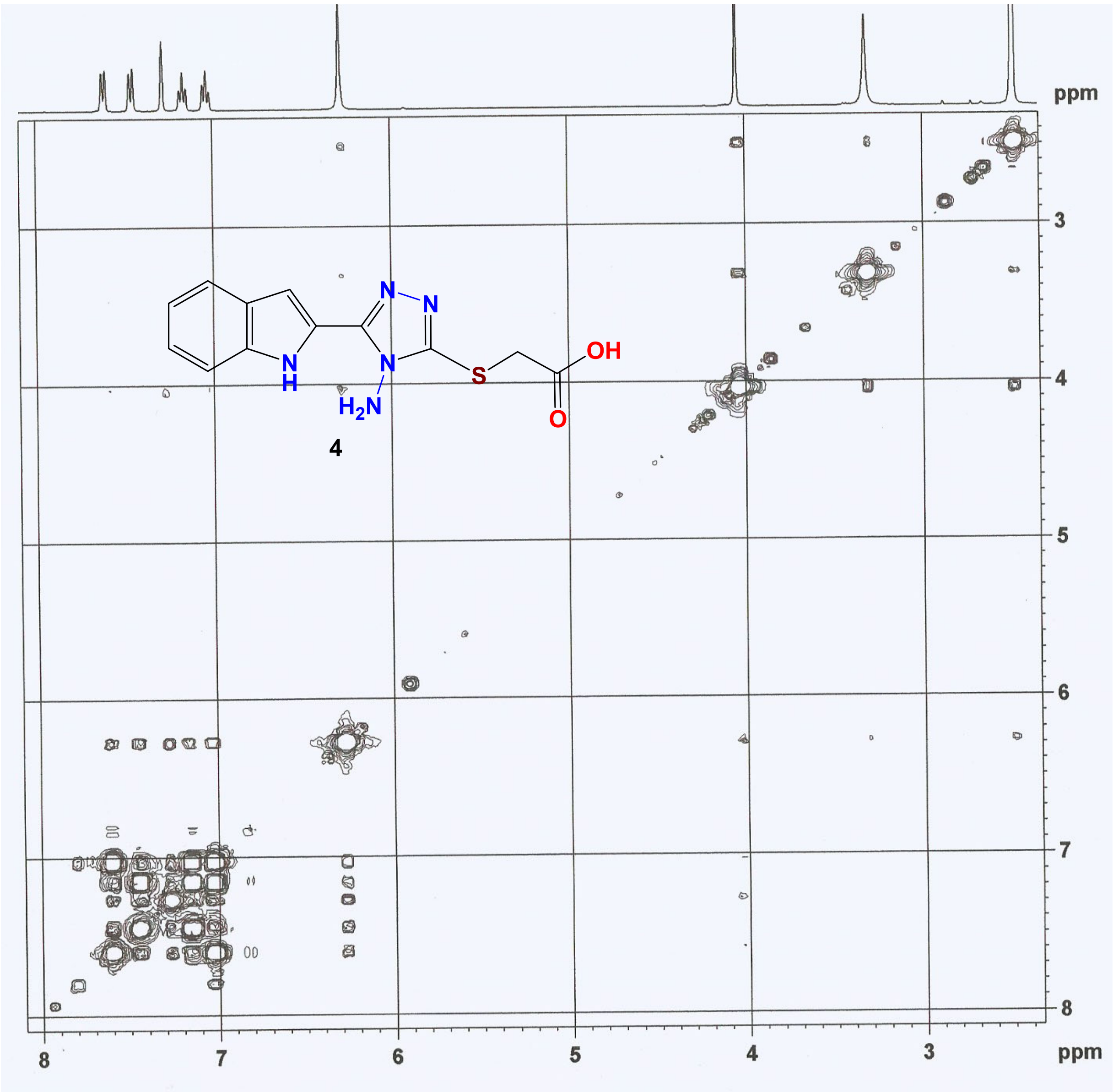

Figure S4. COSY of **4**

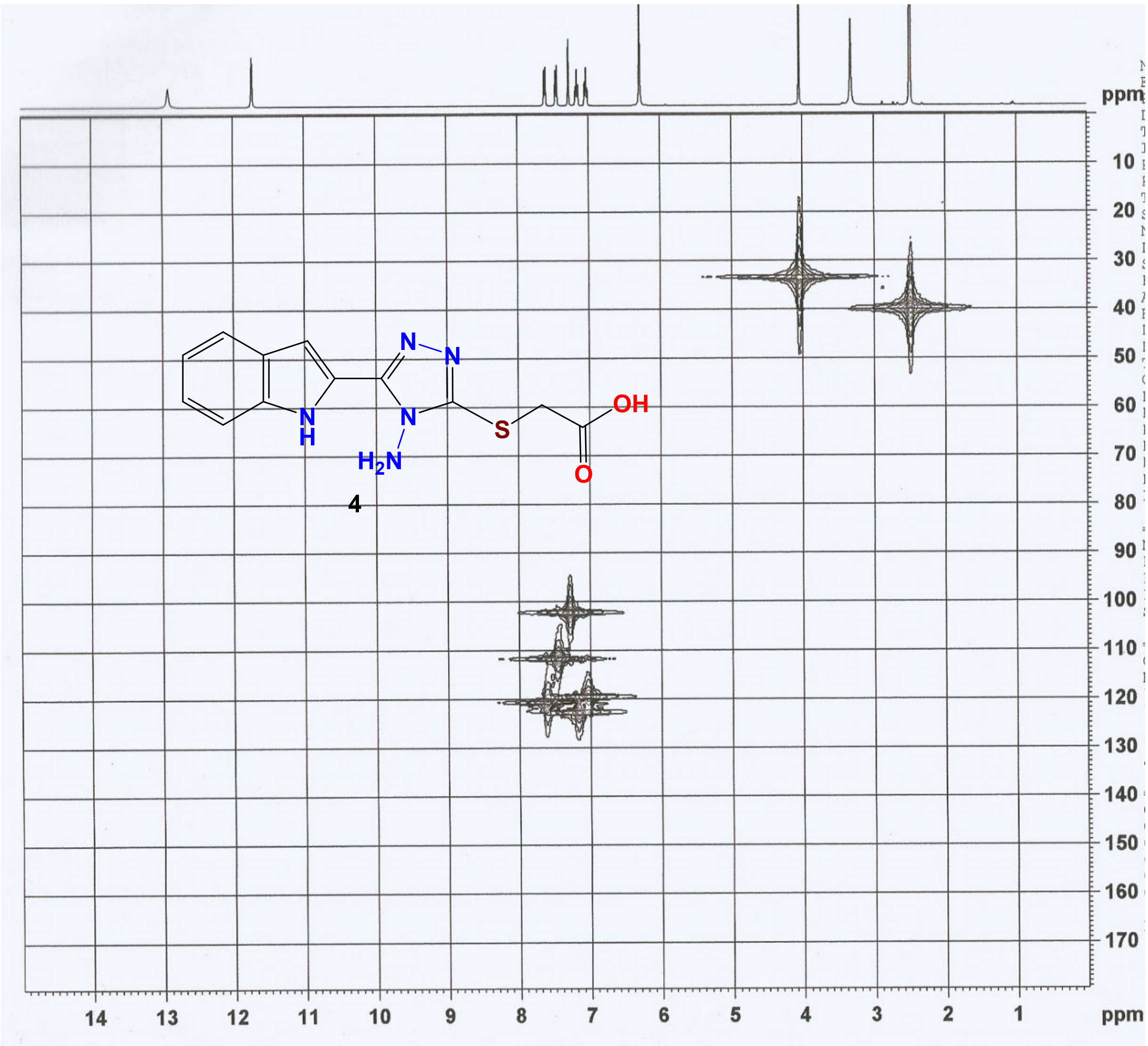

Figure S5. HMQC of **4**

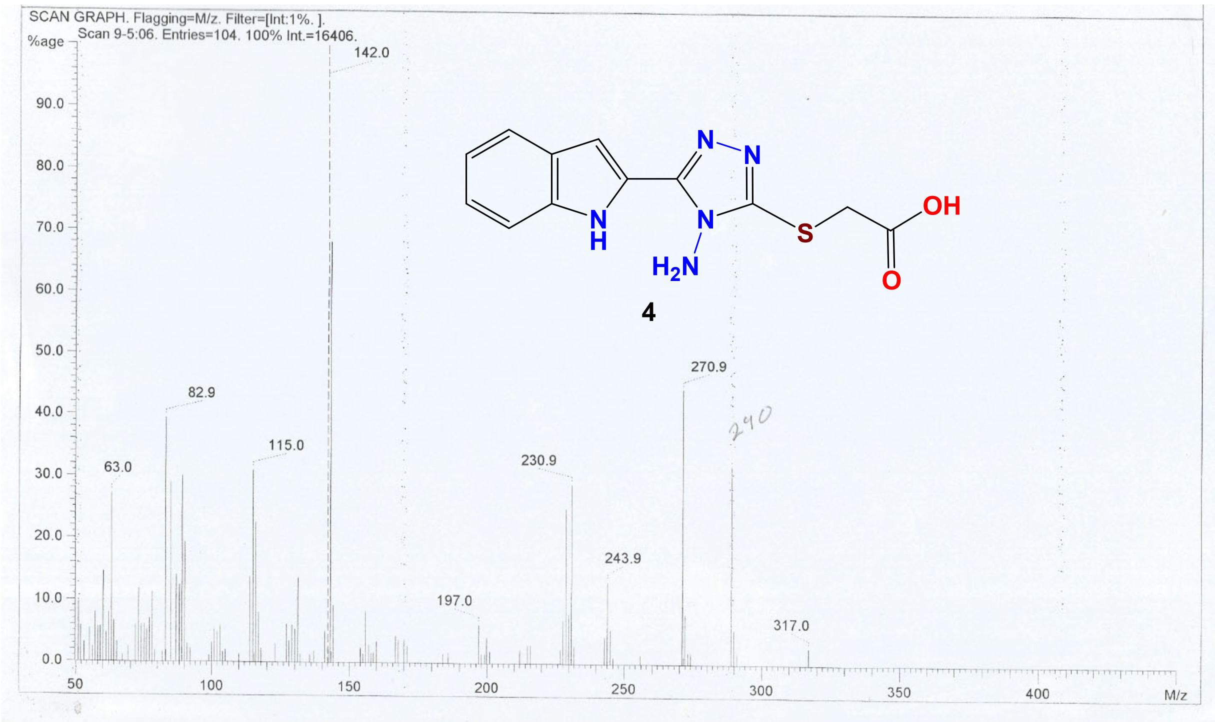

Figure S6. EIMS of **4**

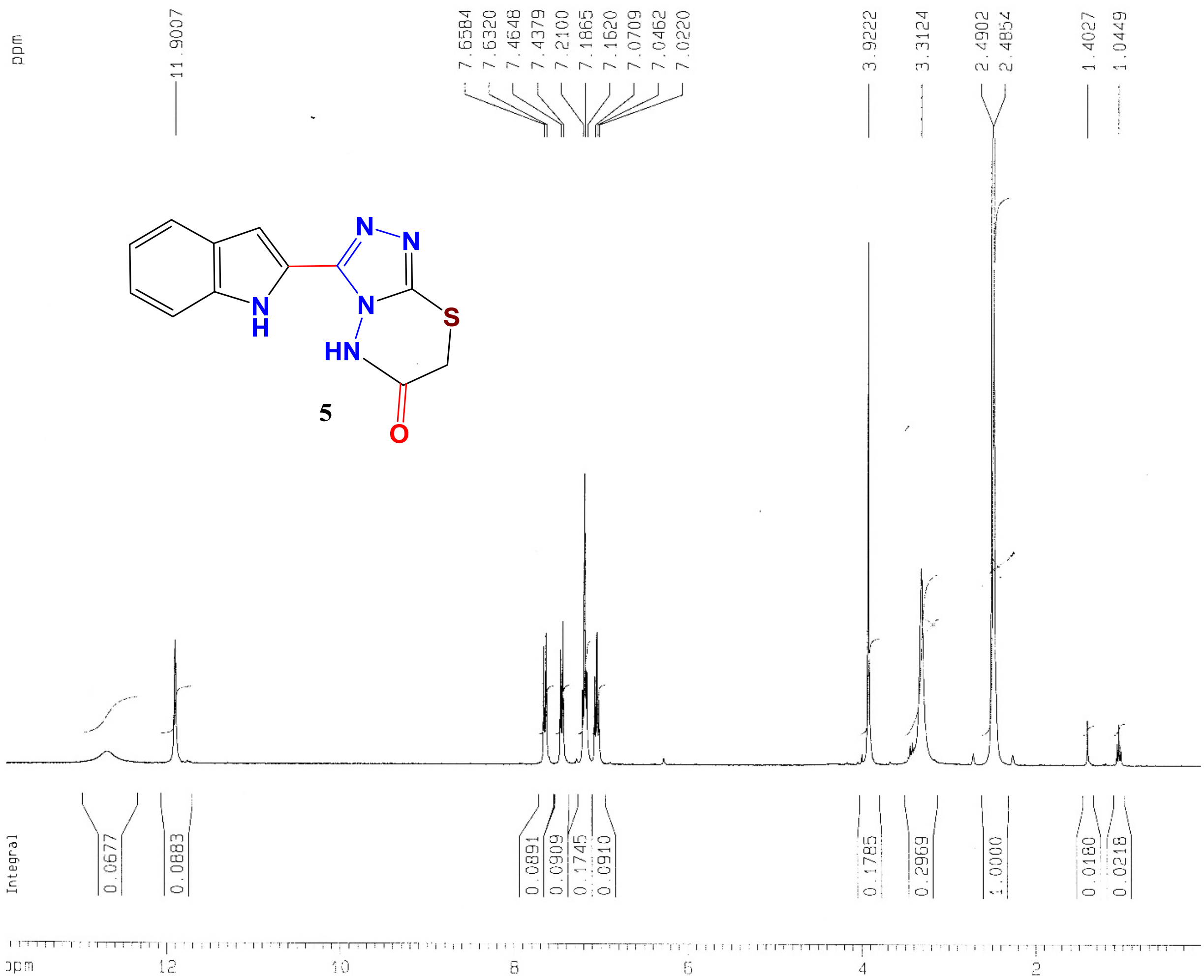

Figure S7. ^{1}H NMR of 5

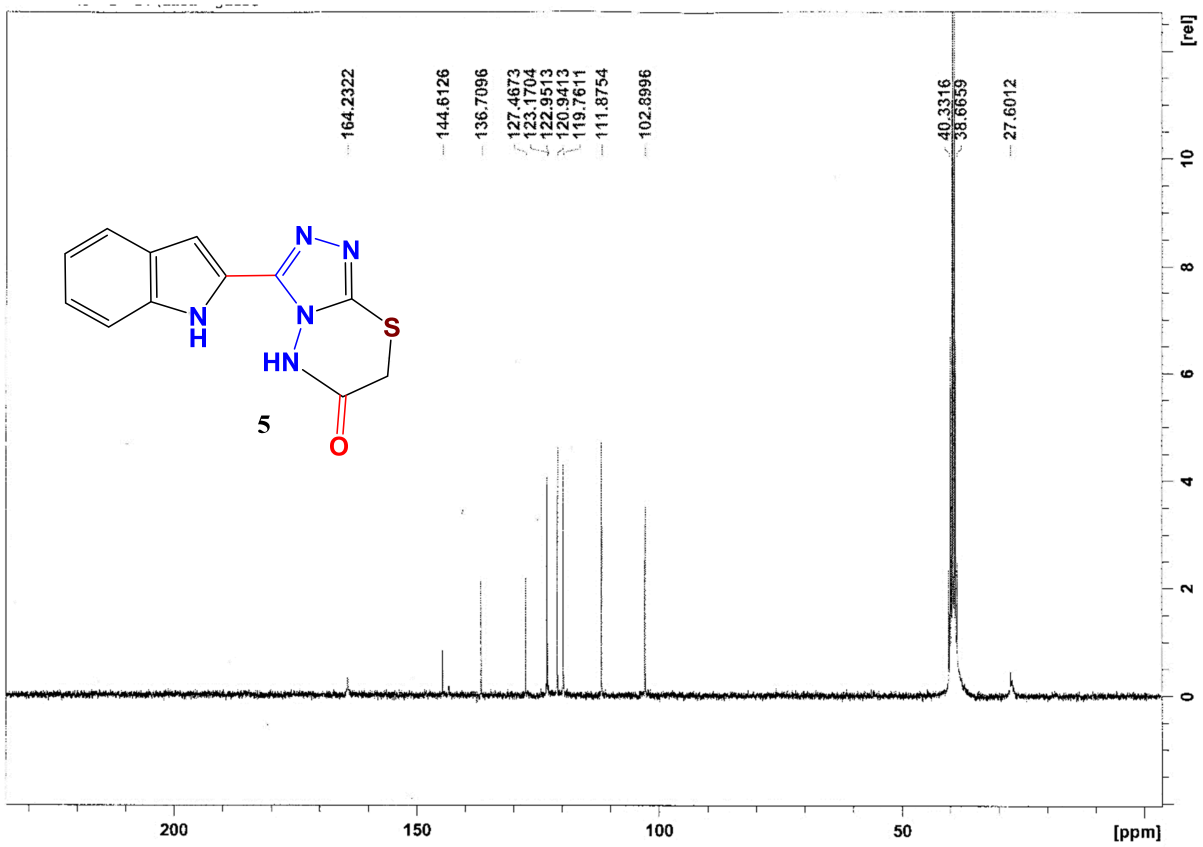

Figure S8. ^{13}C NMR of **5**

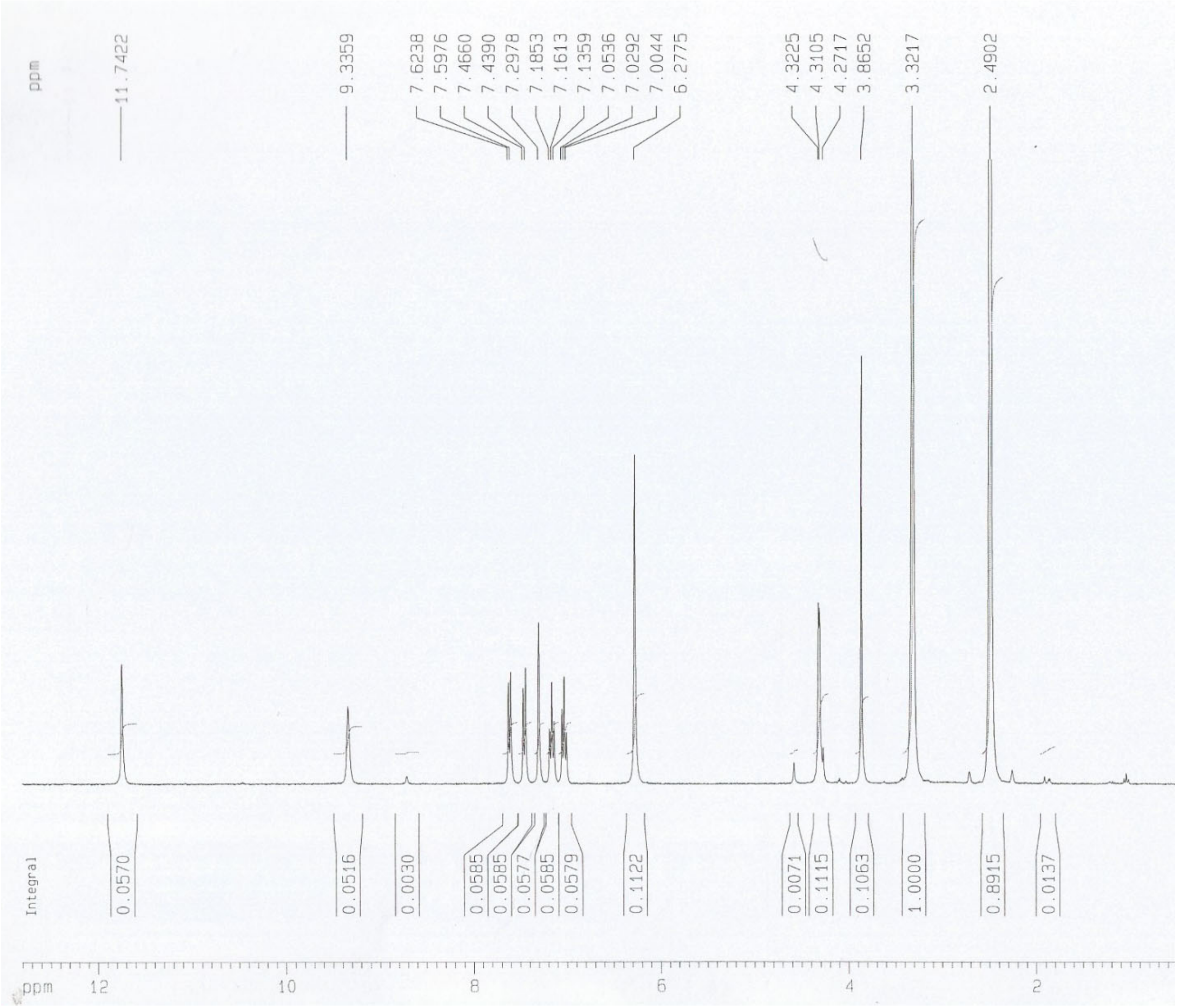

Figure S9. ^{1}H NMR of **6**

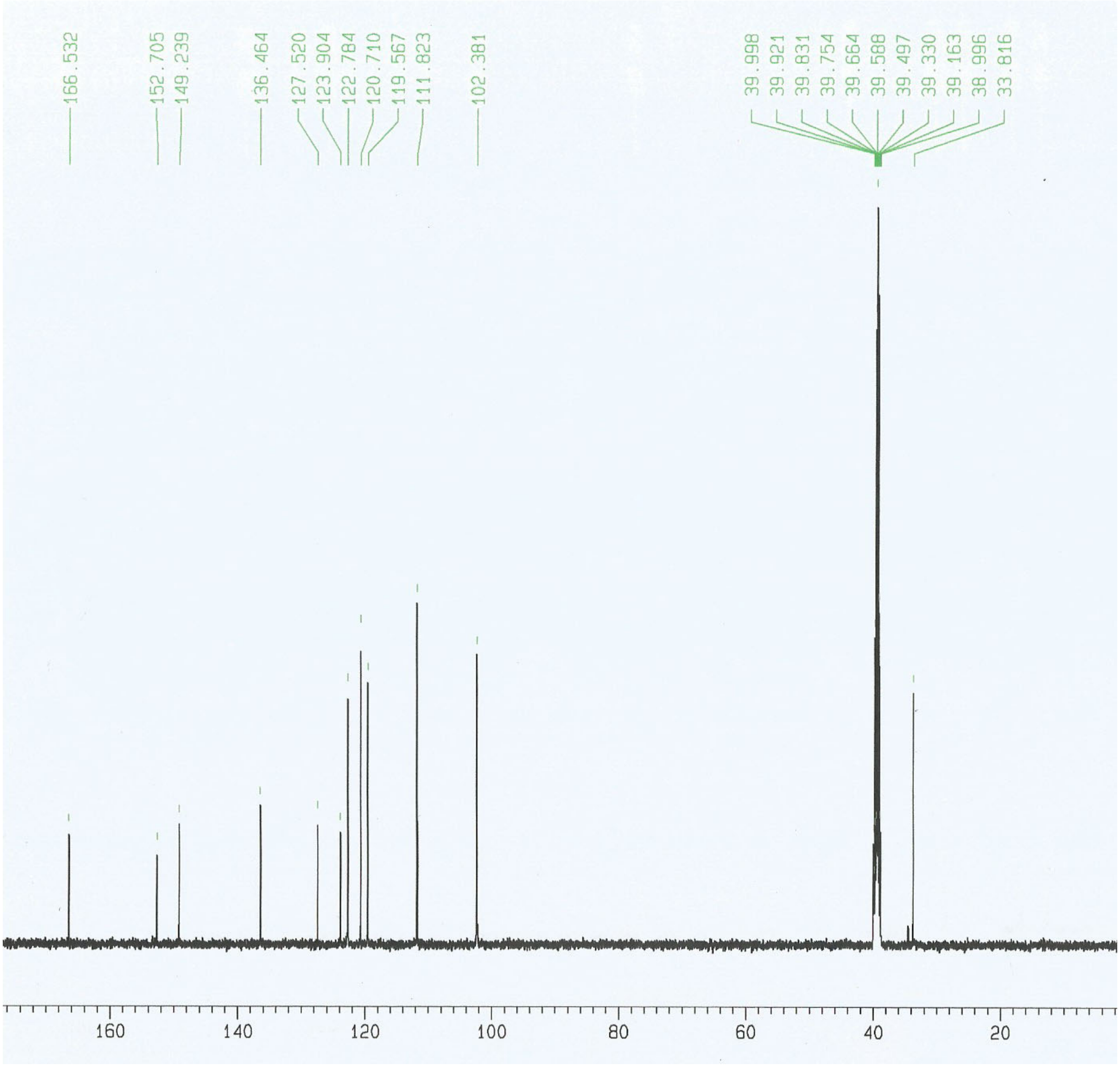

Figure S10. ^{13}C NMR of **6**

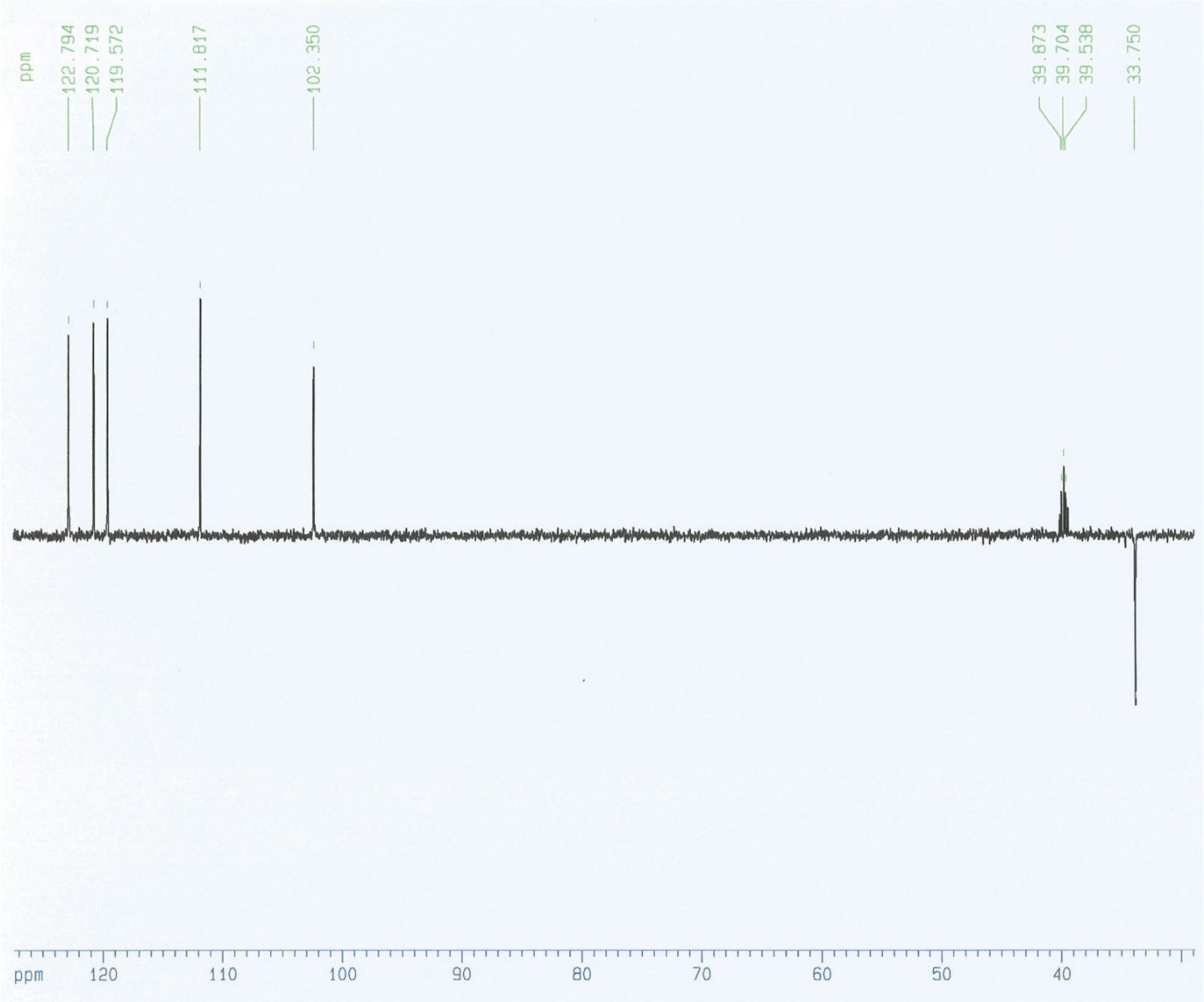

Figure S11. DEPT135 NMR of **6**

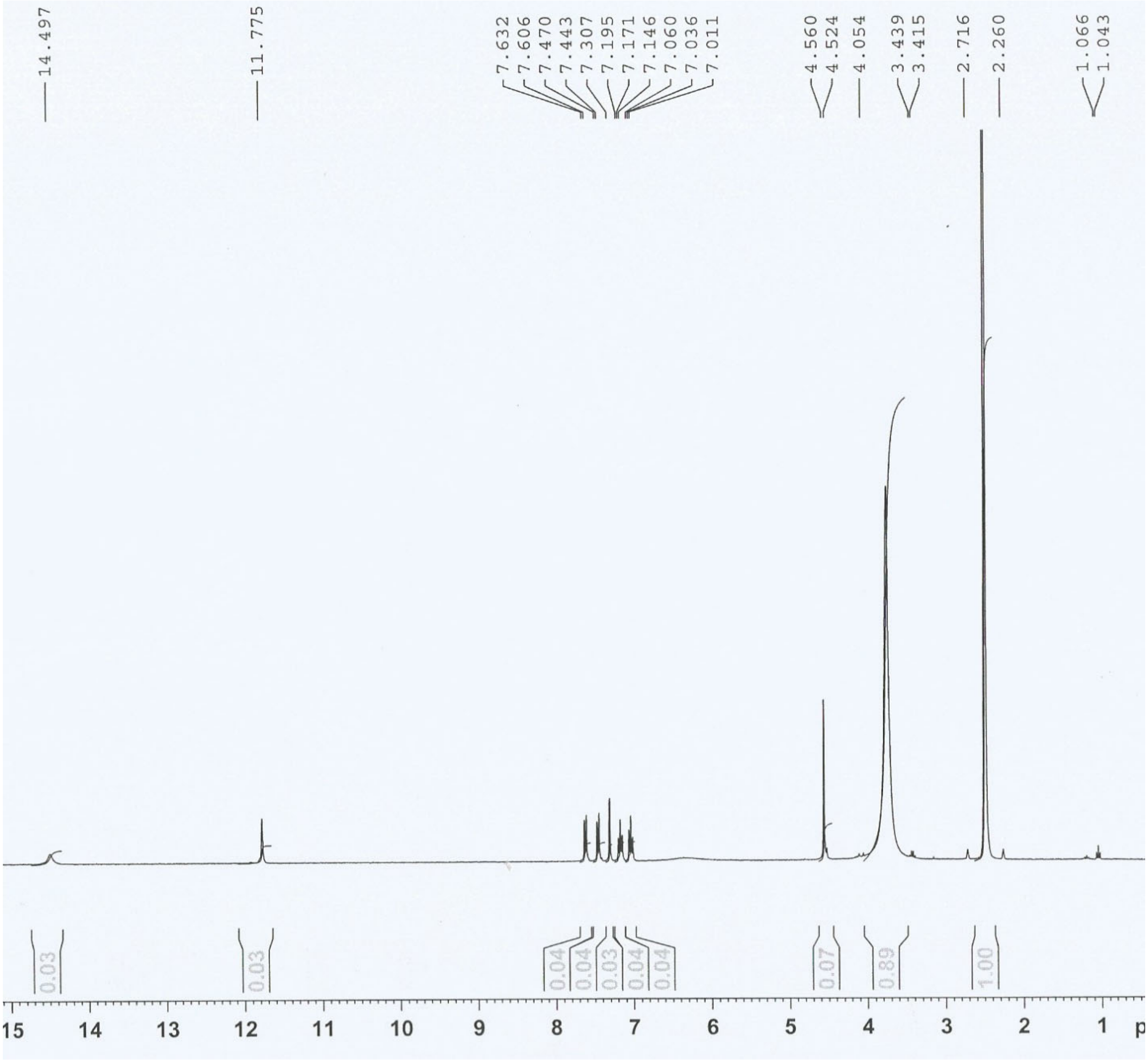

Figure S12. ^{1}H NMR of **7**

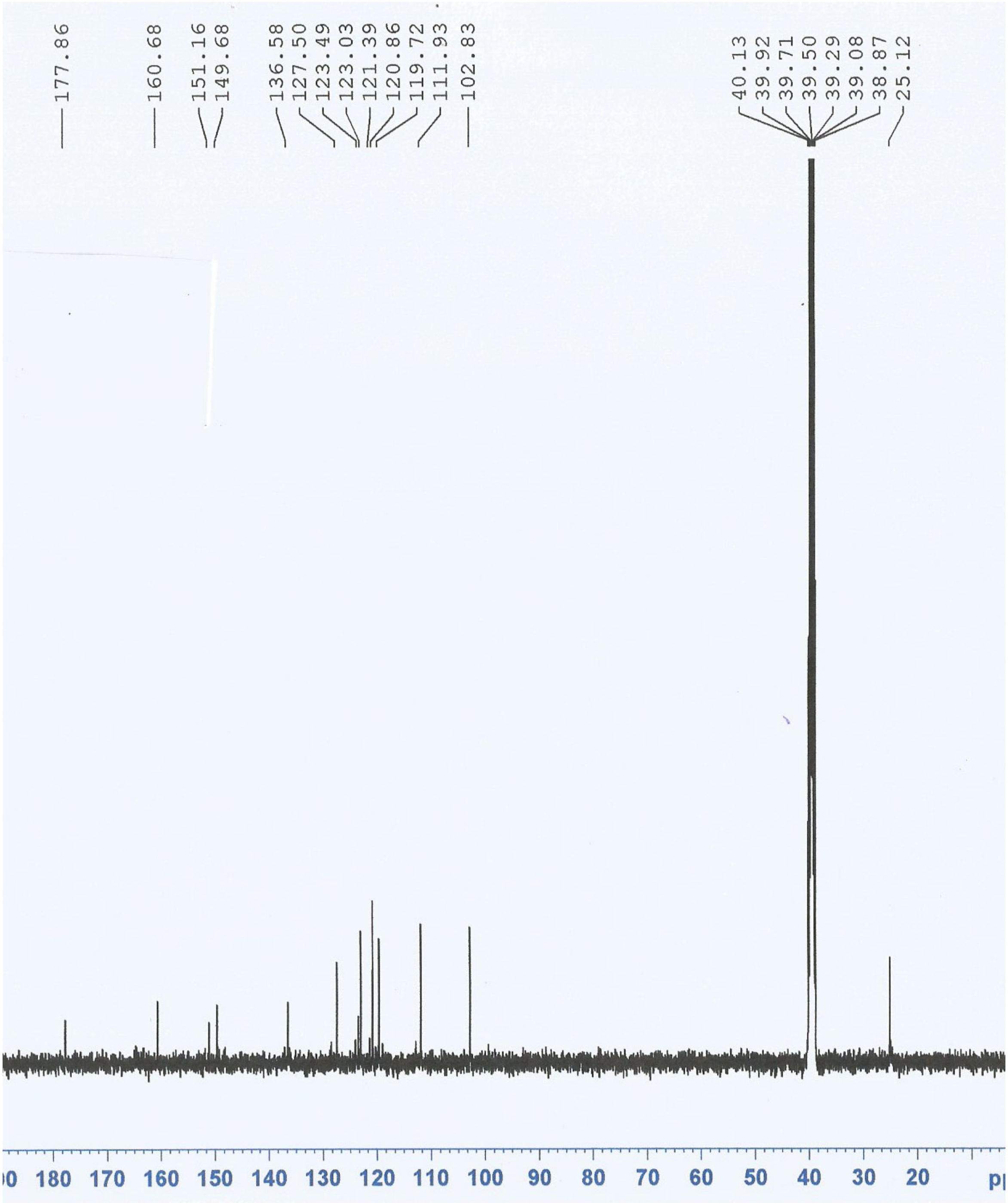

Figure S13. ^{13}C NMR of **7**

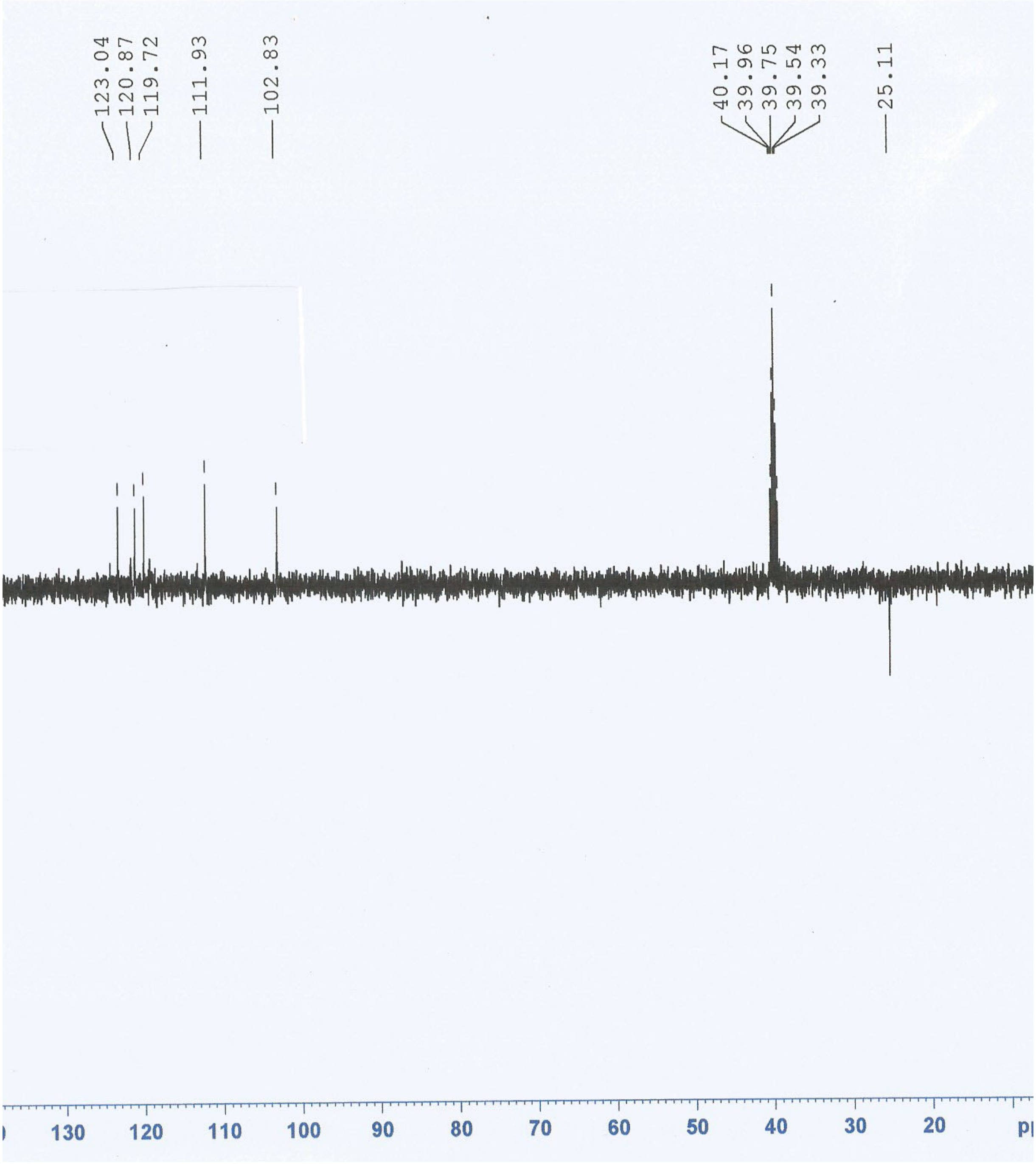

Figure S14. DEPT135 NMR of **7**

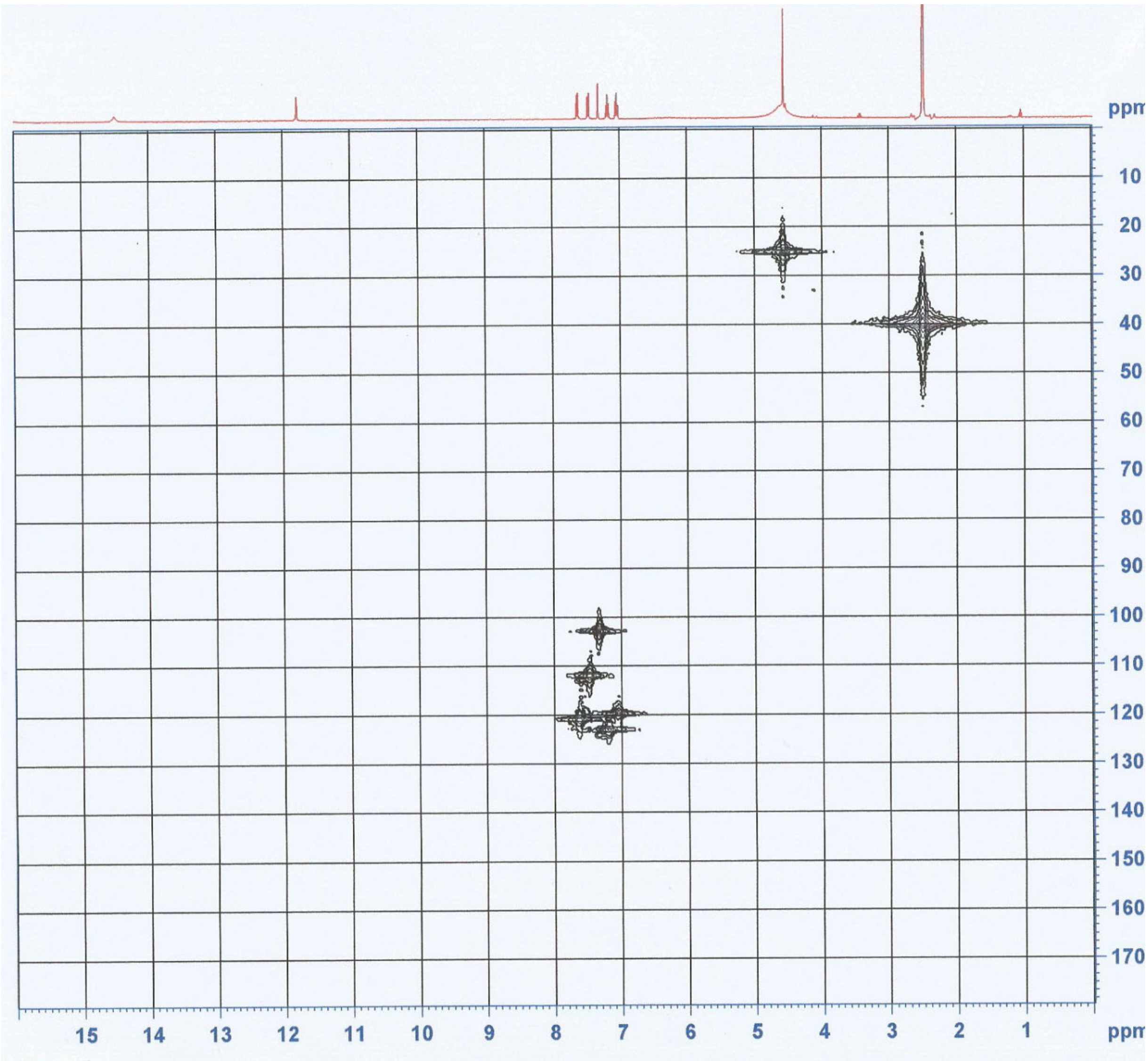

Figure S15. HMQC NMR of **7**

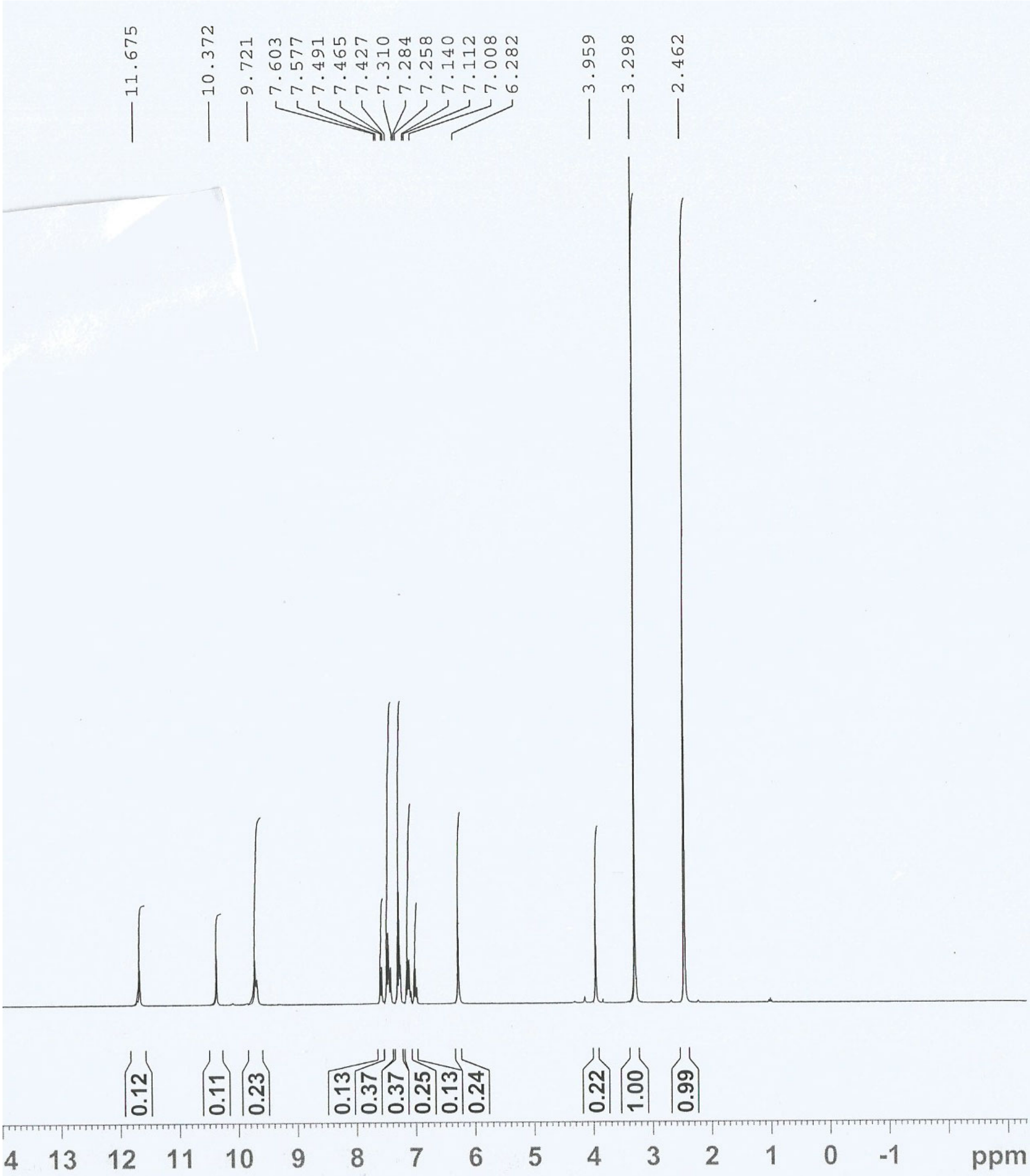

Figure S16. ^{1}H NMR of **9**

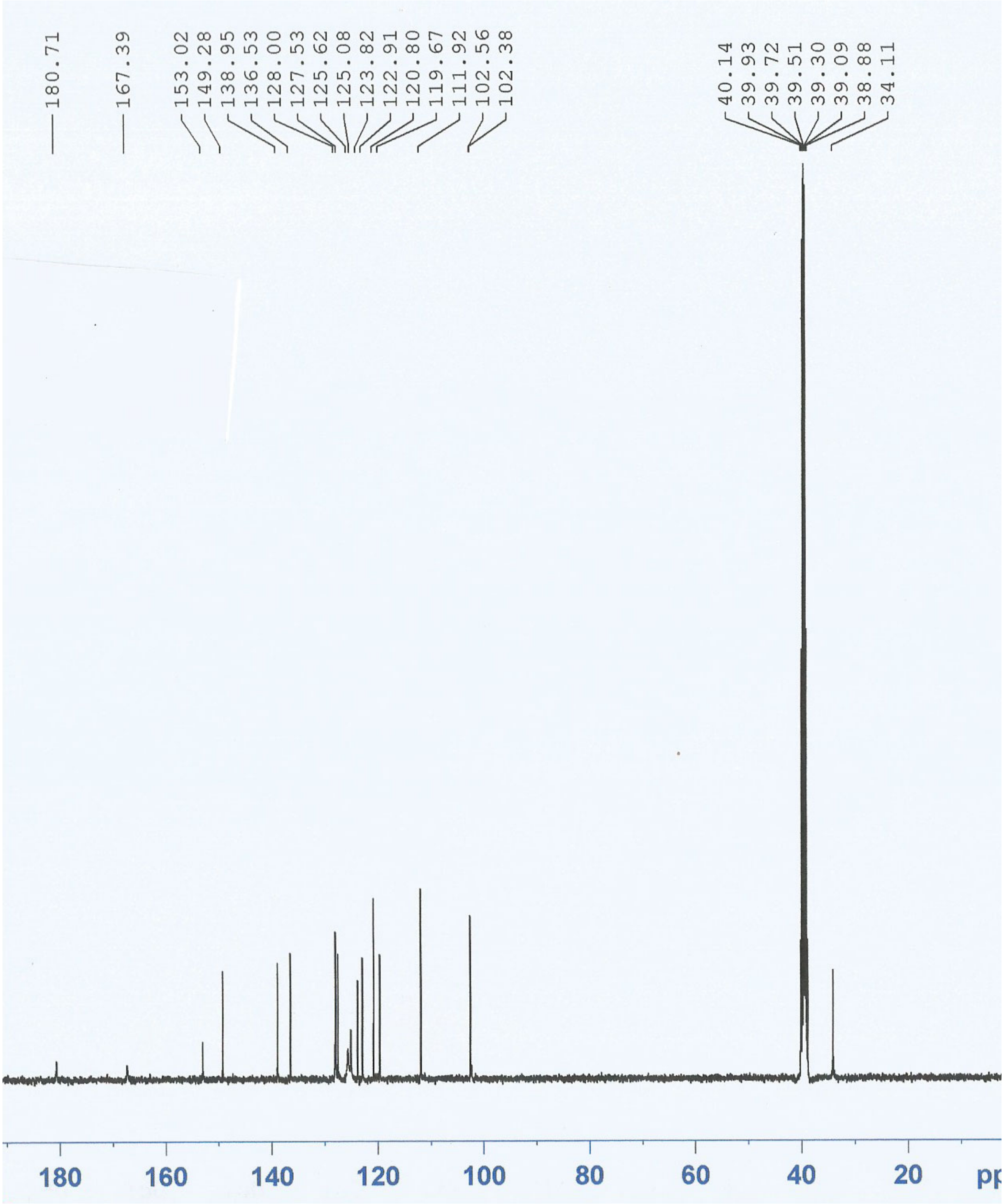

Figure S17. ^{13}C NMR of **9**

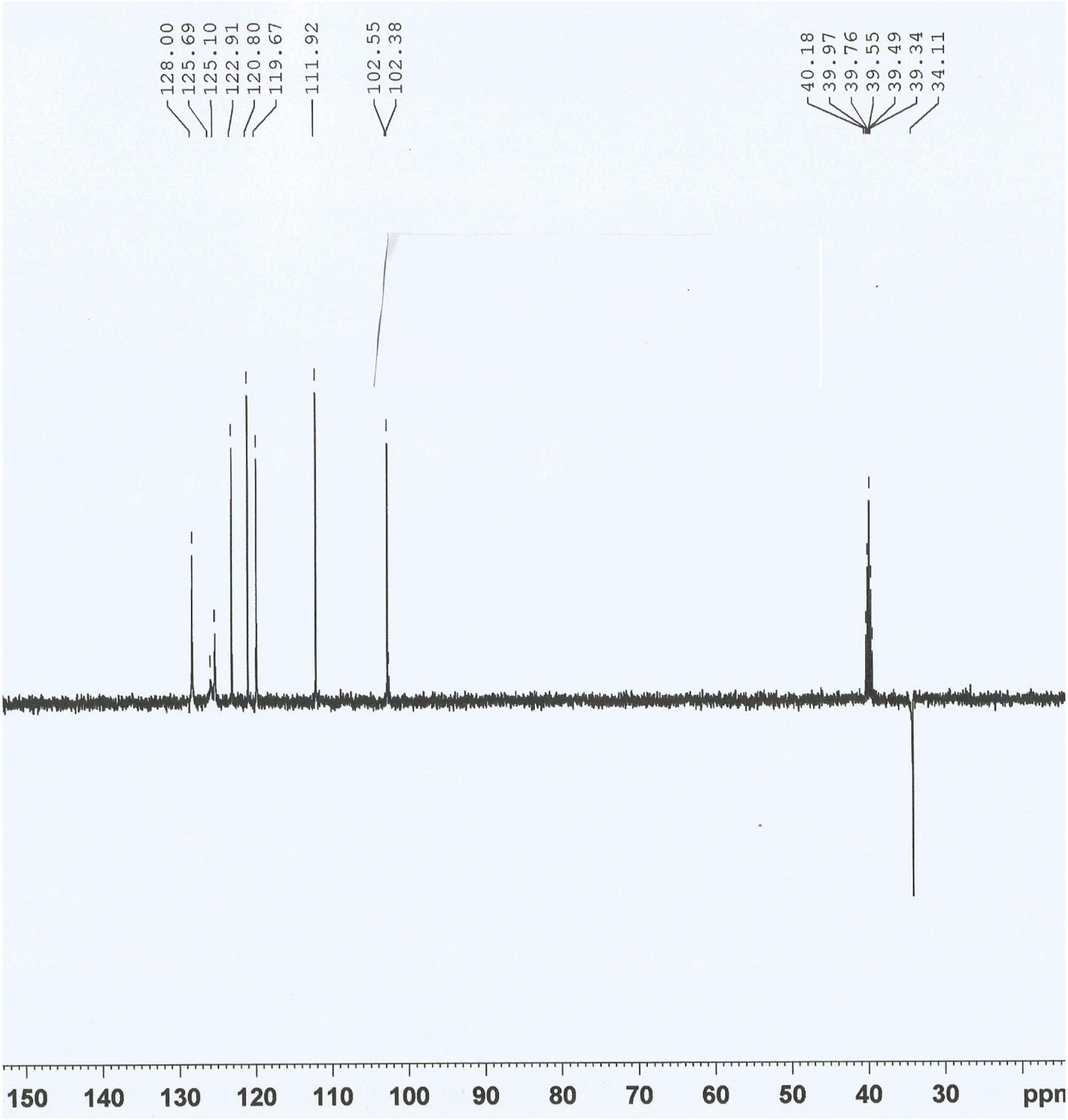

Figure S18. DEPT of **9**

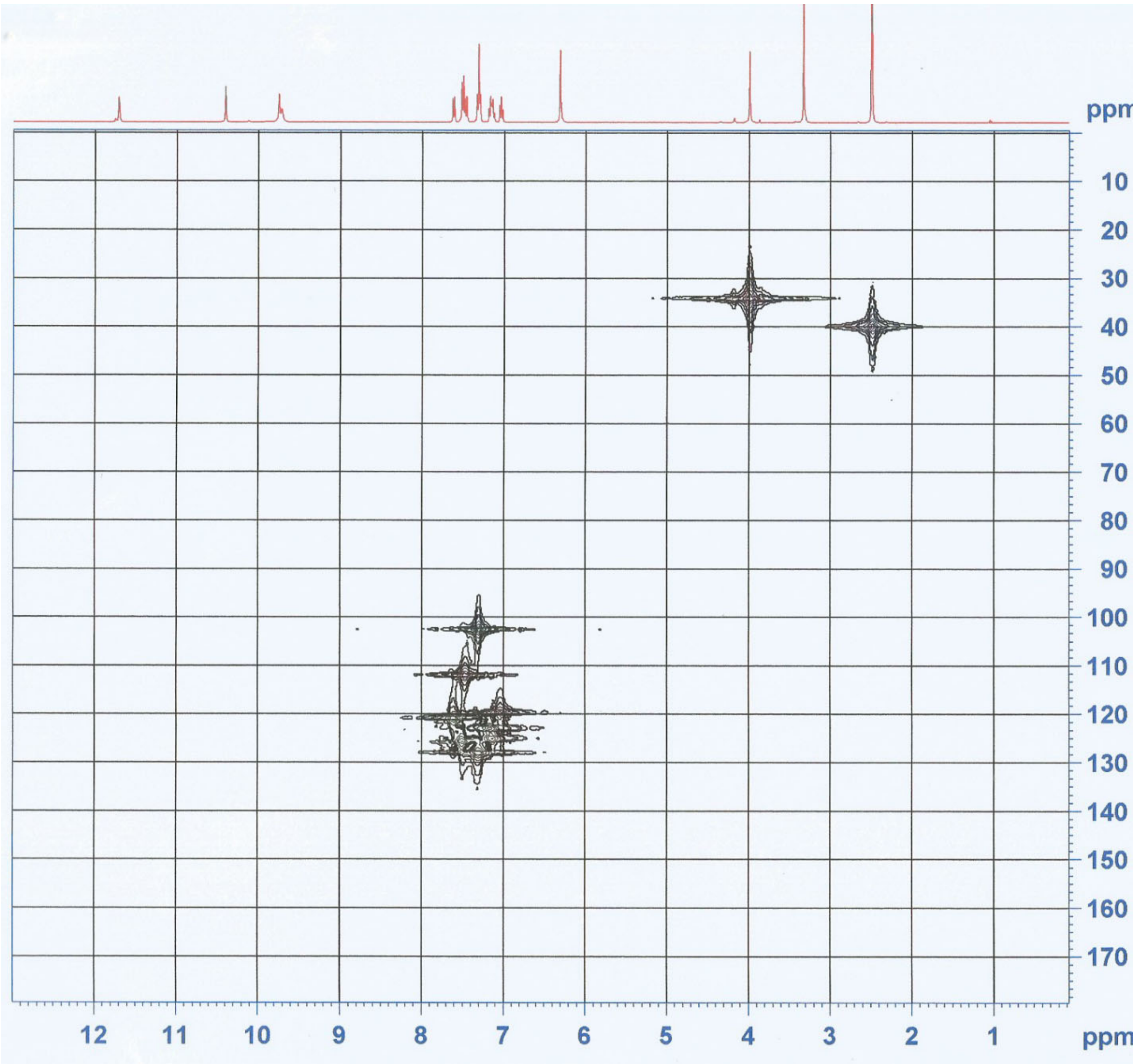

Figure S19. HMQC of **9**

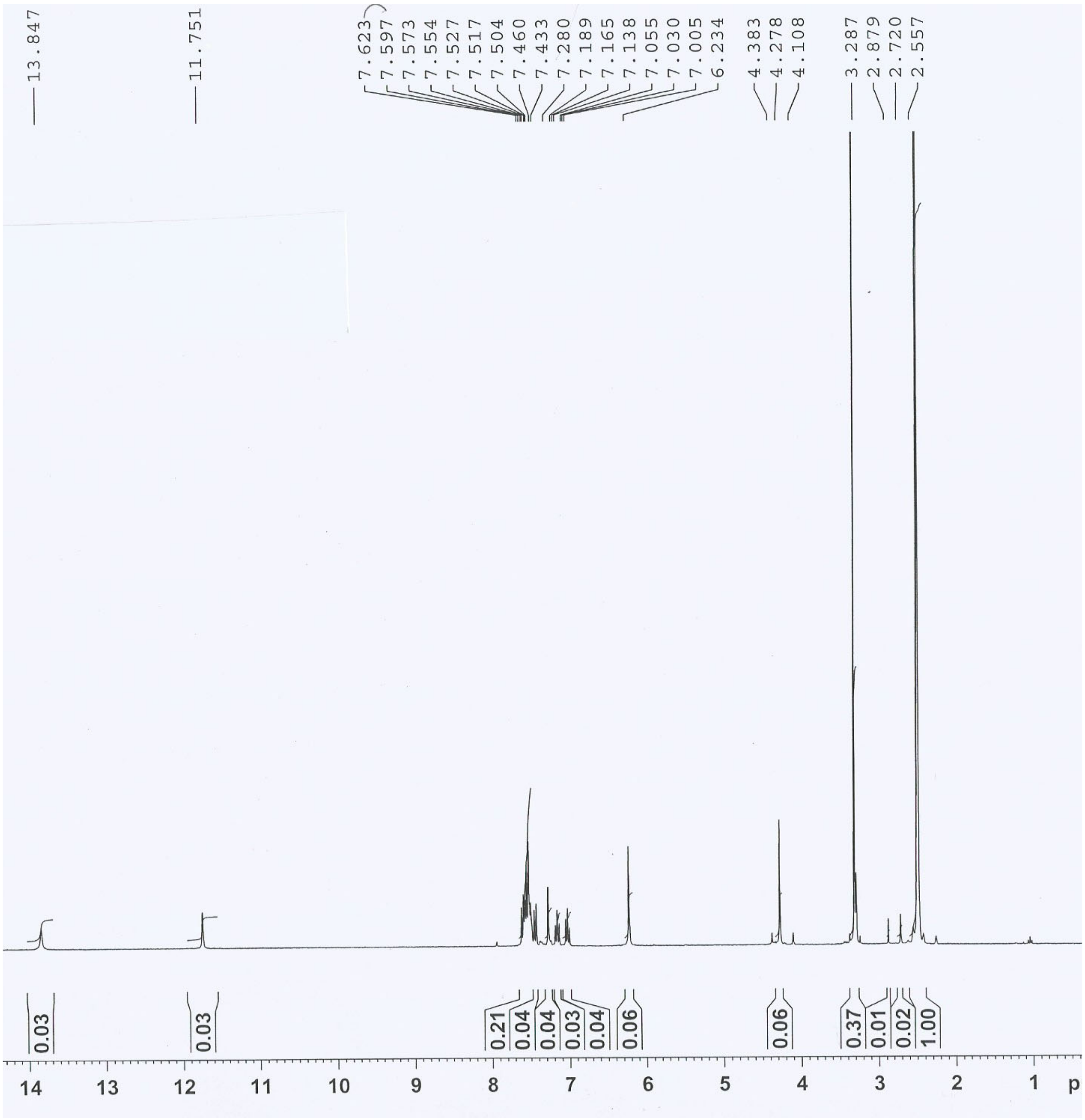

Figure S20. ^{1}H NMR of **10**

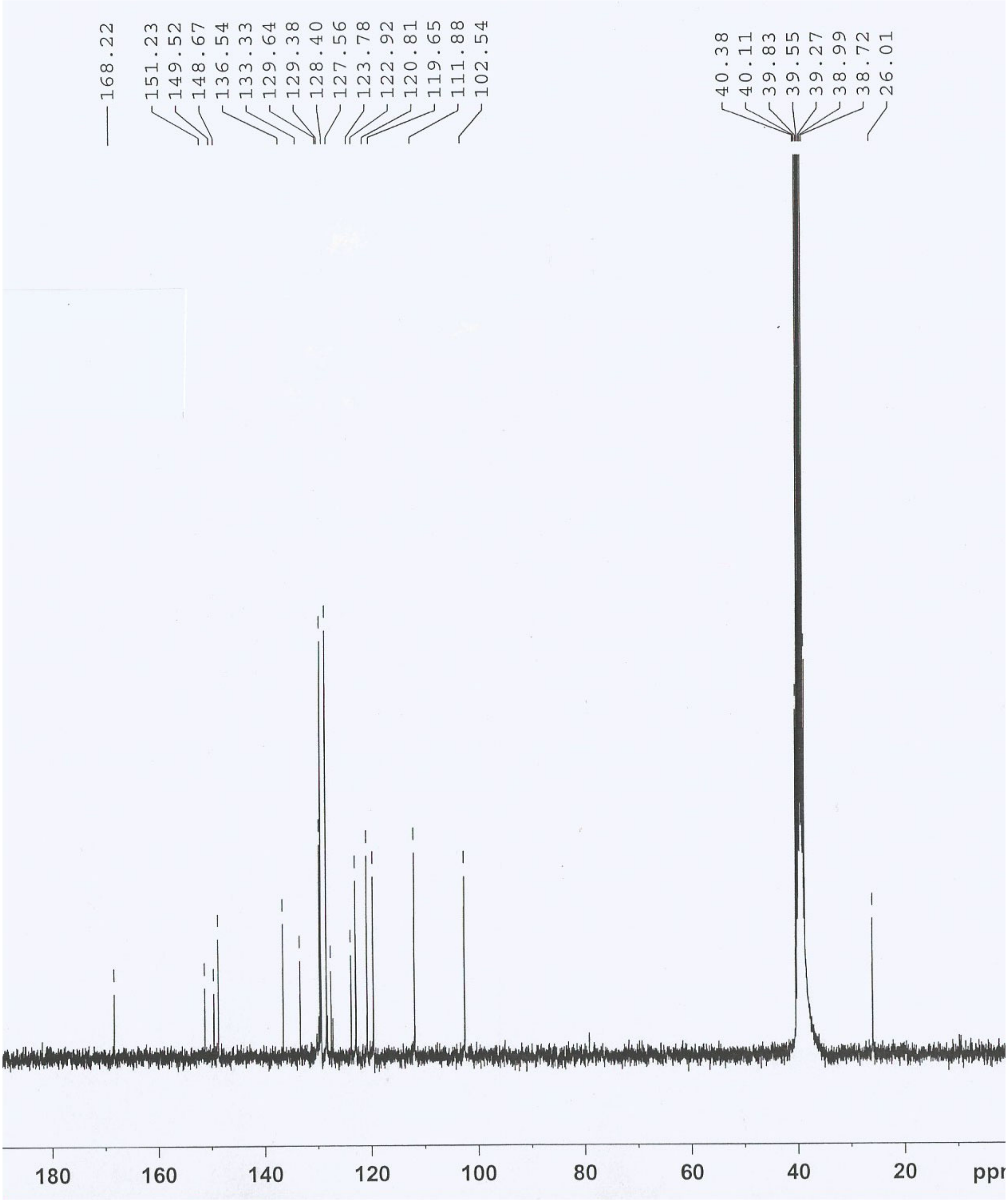

Figure S21. ^{13}C NMR of **10**

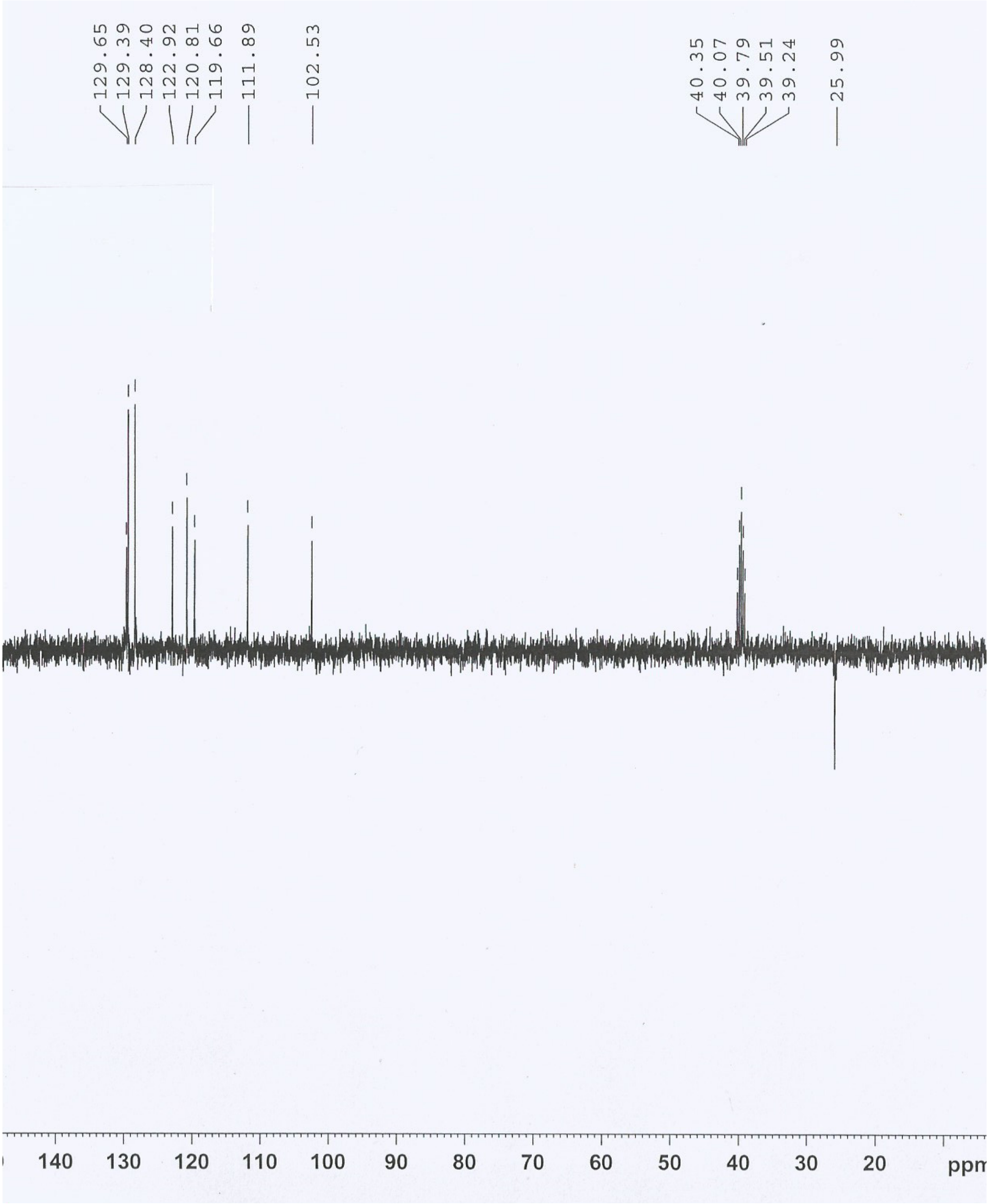

Figure S22. DEPT of **10**

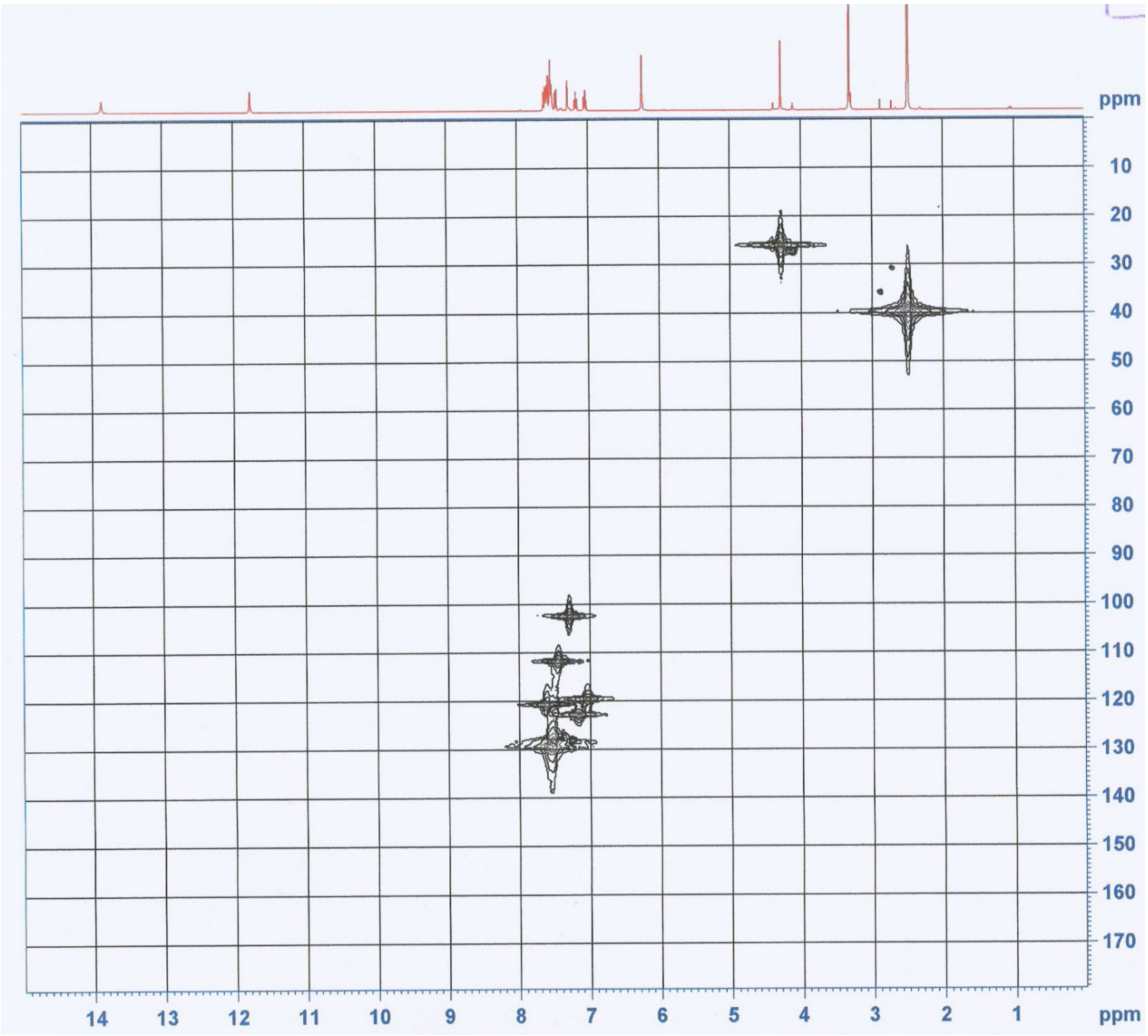

Figure S23. HMQC of **10**

The docking interaction of hit compounds 4 and 9 with Bax, Bcl2, and Caspase-3

In the following figures:

B. represents the 3D interaction.

C. represents the compound inside the protein pocket.

D. represents 3D compound on the surface of protein.

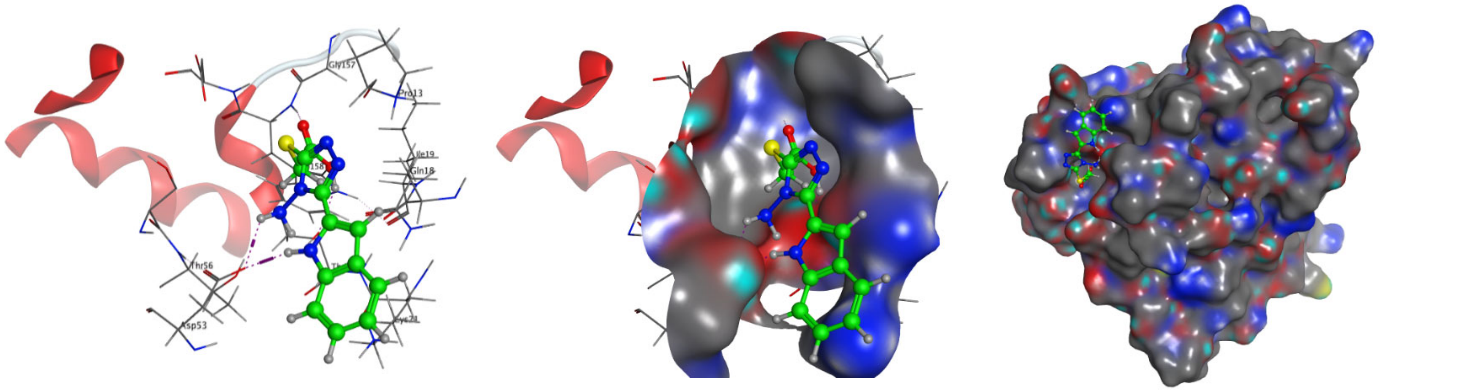

B. 3D isolated of **4** with Bax　　C. 3D of **4** with Bax inside the pocket　　D. 3D of **4** on the surface of protein

Figure S24. Interaction of Compound **4** with Bax.

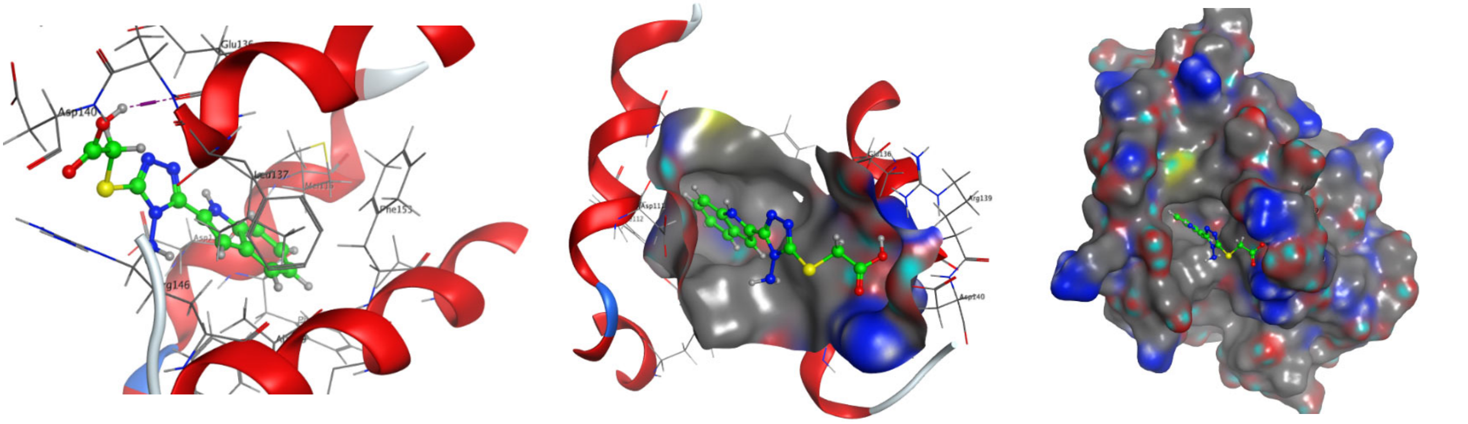

B. 3D isolated of **4** with Bcl2　　C.3D of **4** with Bcl2 inside the pocket　　D. 3D of **4** on the surface of protein

Figure S25. Interaction of compound **4** with Bcl2.

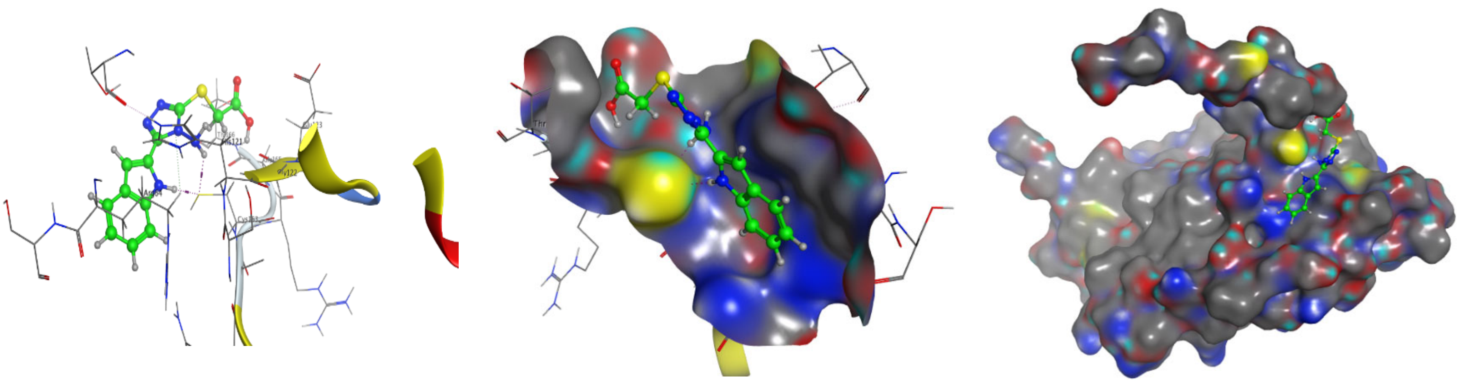

B. 3D isolated of **4** with Caspase-3　　C. 3D of **4** with Caspase-3 inside the pocket　　D. 3D of **4** on the surface of protein

Figure S26. Interaction of compound **4** with Caspase-3.

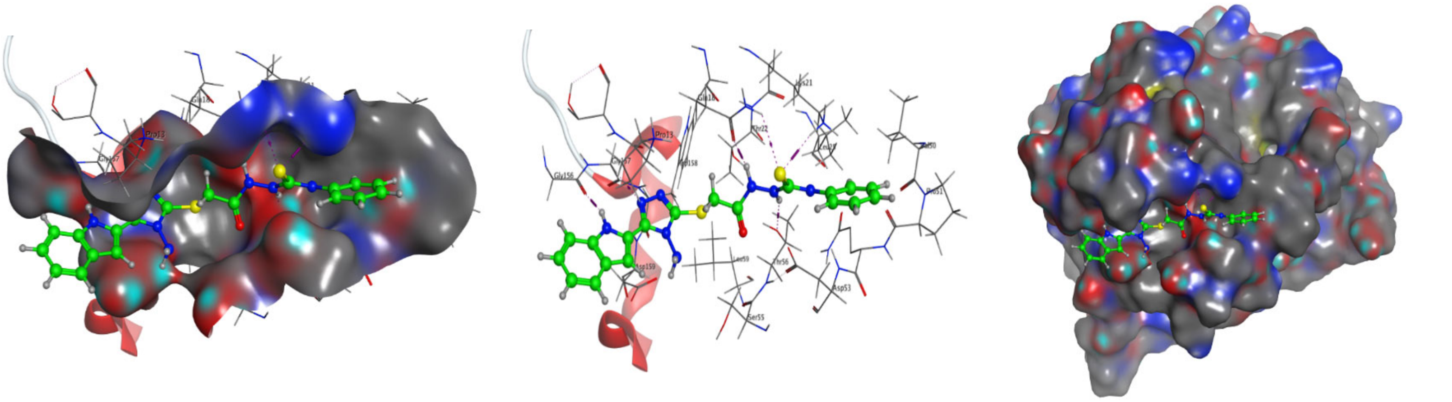

B. 3D isolated of **9** with Bax

C. 3D of **9** with Bcl2 inside the pocket

D. 3D of **9** on the surface of protein

Figure S27. Interaction of Compound **9** with Bax.

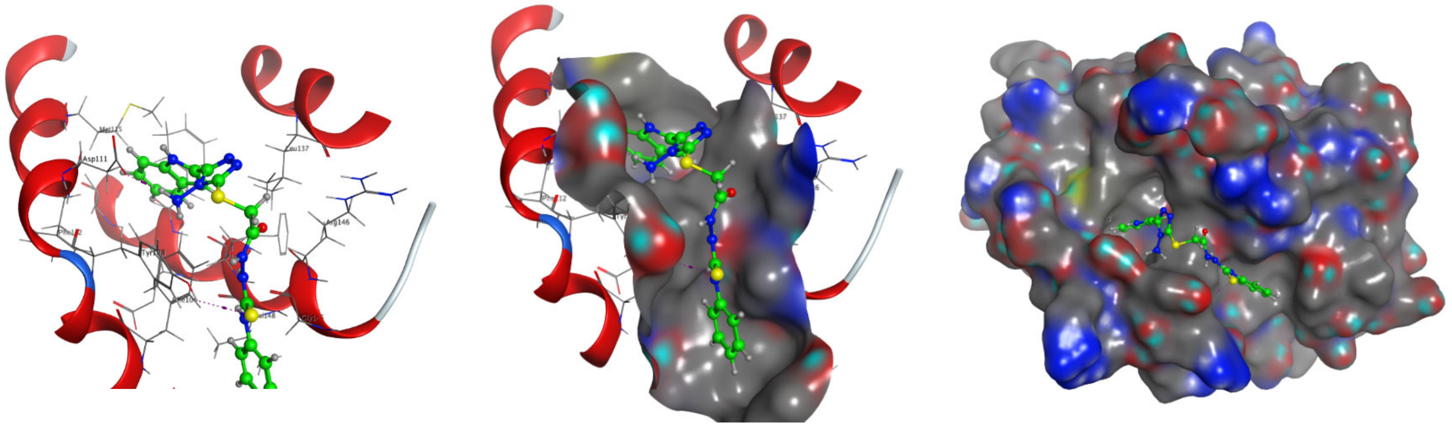

B. 3D isolated of **9** with Bcl2

C. 3D of **9** with Bcl2 inside the pocket

D. 3D of **9** on the surface of protein

Figure S28. Interaction of compound **9** with Bcl2.

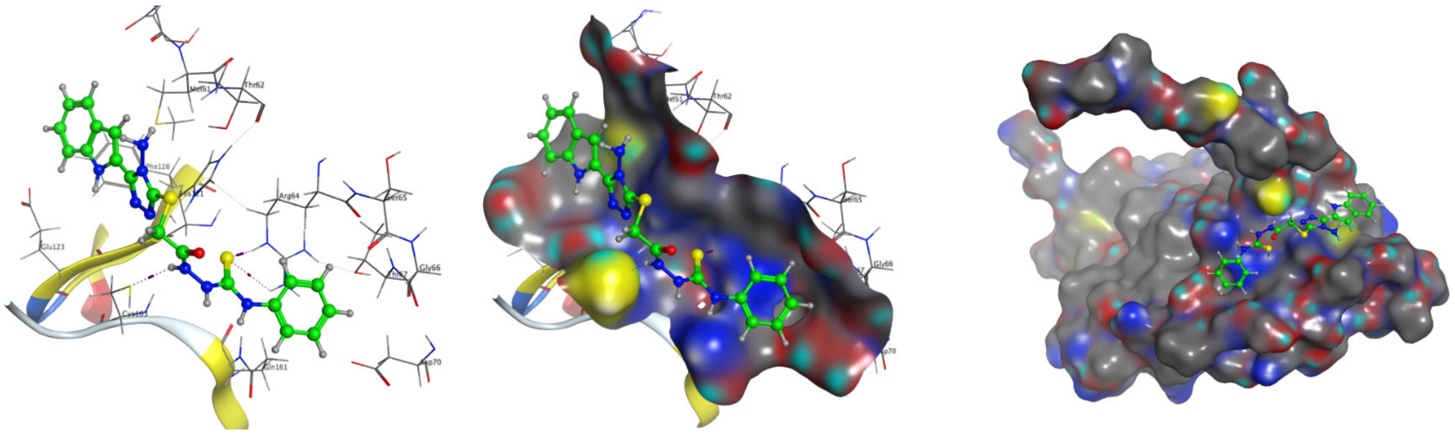

B. 3D isolated of **9** with Caspase-3

C. 3D of **9** with Caspase-3 inside the pocket

D. 3D of **9** on the surface of protein

Figure S29. Interaction of compound **9** with Caspase-3.

Cytotoxic activity of the synthesized compounds against the HepG2, MCF-7 cell lines and VEGFR-2 (run in triplicate)

Table S1. HepG2 IC$_{50}$ values (triplicate measurements) and calculated mean ± SD (n = 3).

Compound	1st value	2nd value	3rd value	**Mean±SD (n=3)**
1	1.8044	1.7947	1.8000	1.8 ± 0.0028
2	0.9643	0.9597	0.9652	0.96 ± 0.0017
3	2.0922	2.0799	2.0698	2.1 ± 0.0065
4	0.2755	0.2719	0.2687	0.27 ± 0.002
5	0.3147	0.3174	0.3194	0.32 ± 0.0014
6	1.5177	1.5770	1.4703	1.5 ± 0.031
7	5.6807	5.9368	5.8112	5.8 ± 0.074
9	0.4100	0.4104	0.4088	0.41 ± 0.00048
10	1.1949	1.1820	1.1868	1.2 ± 0.0038
Doxo.	0.1577	0.1578	0.1577	0.16 ± 0.000033

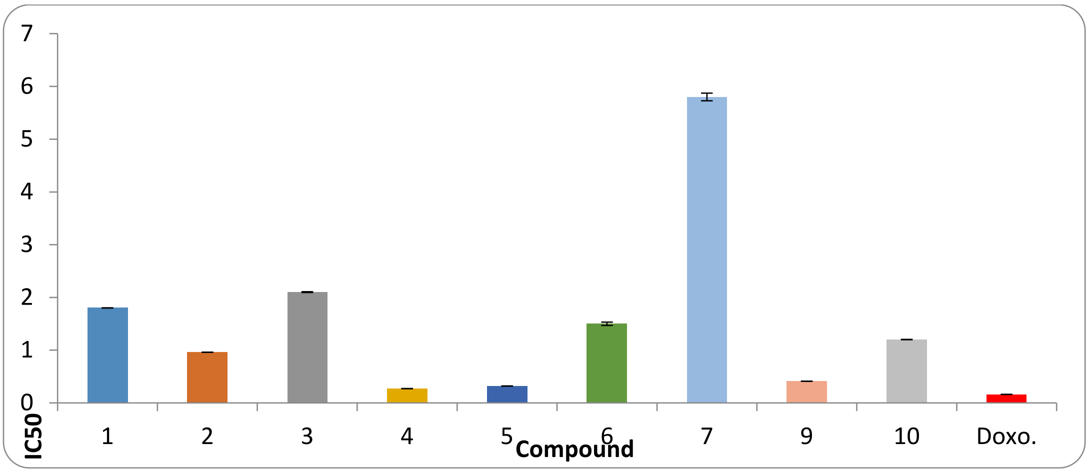

Figure S30. HepG2 IC$_{50}$

Table S2. MCF-7 IC$_{50}$ values (triplicate measurements) and calculated mean ± SD (n = 3).

Compound	1st value	2nd value	3rd value	**Mean±SD (n=3)**
1	0.2117	0.2110	0.2119	0.21 ± 0.00027
2	0.9582	0.9488	0.9545	0.95 ± 0.0027
3	0.3467	0.3583	0.3510	0.35 ± 0.0034
4	0.9621	0.9686	0.9695	0.97 ± 0.0023
5	0.2349	0.2354	0.2367	0.24 ± 0.00054
6	0.8181	0.8192	0.8184	0.82 ± 0.00033
7	4.6269	4.6353	4.6482	4.6 ± 0.0062
9	0.4910	0.4867	0.4931	0.49 ± 0.0019
10	0.2468	0.2459	0.2455	0.25 ± 0.00038
Doxo.	0.1031	0.1009	0.1015	0.1 ± 0.00066

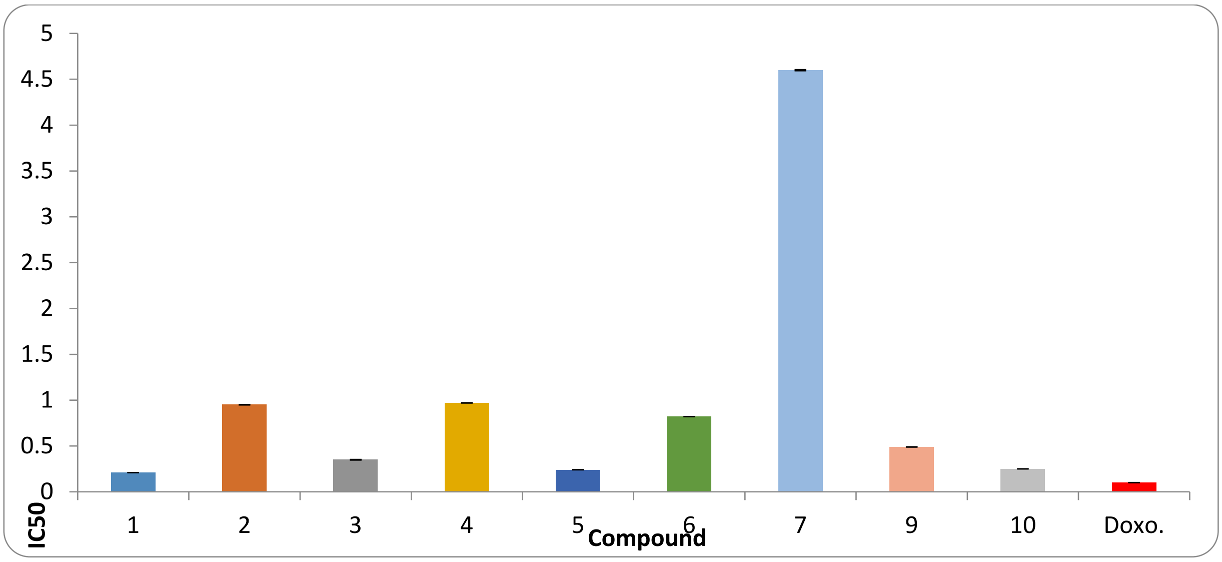

Figure S31. MCF-7 IC_{50}

Table S3. VEGFR‑2 IC_{50} values (triplicate measurements) and calculated mean ± SD (n = 3).

Compound	1st value	2nd value	3rd value	**Mean±SD (n=3)**
1	2.298	2.325	2.285	2.3 ± 0.012
4	0.583	0.591	0.589	0.59 ± 0.004
5	2.375	2.417	2.378	2.4 ± 0.014
9	1.279	1.203	1.207	1.2 ± 0.025
10	2.308	2.341	2.366	2.3 ± 0.017
Sunitinib	0.320	0.322	0.303	0.32 ± 0.006

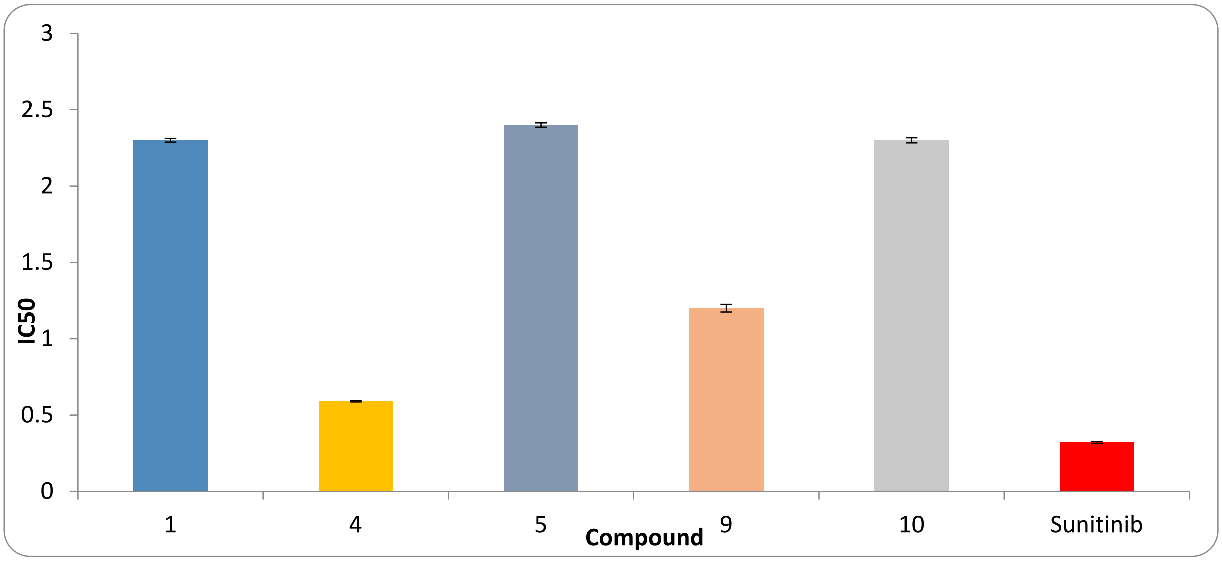

Figure S32. VEGFR-2 IC_{50}

Western blotting

Cell lysates were adjusted to equal protein concentration in PBS, mixed 1:1 (v/v) with 2× Laemmli sample buffer (0.125 M Tris-HCl, pH 6.8; 10% glycerol; 4% SDS; 0.25 M DTT), and heated for 5 min. Proteins were separated by SDS-PAGE and transferred to a PVDF membrane. Membranes were blocked in 5% BSA in Tris-buffered saline (TBS) for 30 min and then incubated with primary antibodies against BAX (Cat. ES1753, ELK Biotechnology, Wuhan, China), Bcl-2 (Cat. ES1758, ELK Biotechnology) and GAPDH (Cat. EM1028, ELK Biotechnology) (all 1:100 dilution) for 1 h. Membranes were washed three times with TBS containing 0.1% Tween-20 (TBST; 5 min each) and incubated with HRP-conjugated secondary antibody (Goat anti-mouse HRP, ELK Biotechnology) for 2 h. After three additional washes with TBST, bands were developed using enhanced chemiluminescence (ECL) reagent and imaged using a ChemiDoc MP system (Biometra, USA).

SDS-PAGE

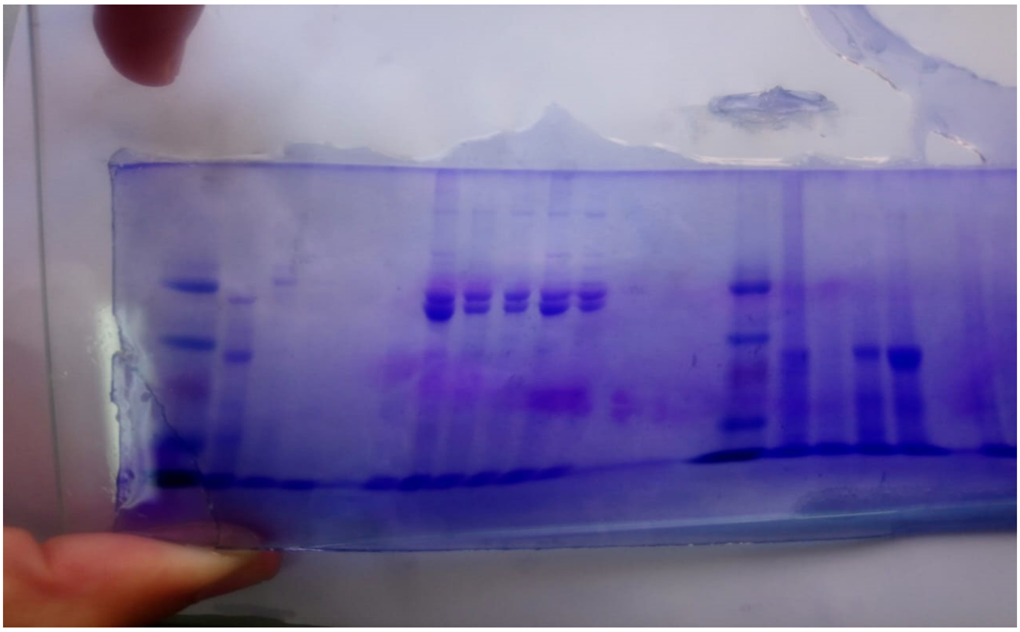

Figure S33. Representative SDS-PAGE gel used for protein separation prior to immunoblotting.

BAX western blot

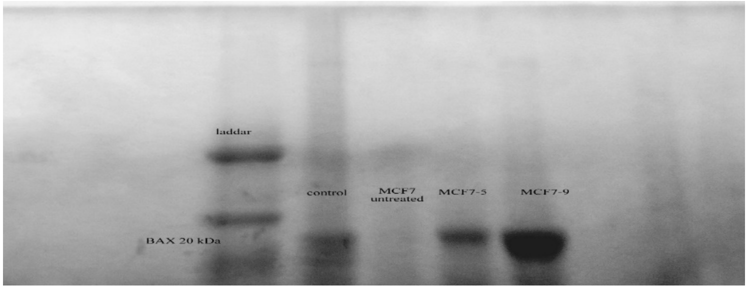

Figure S34. Uncropped western blot showing BAX (20 kDa) expression in control breast cells, untreated MCF-7 cells, and MCF-7 cells treated with compounds 5 and 9.

In this representative blot, treatment with compound 9 shows increased BAX expression relative to untreated MCF-7 cells.

Bcl-2 western blot

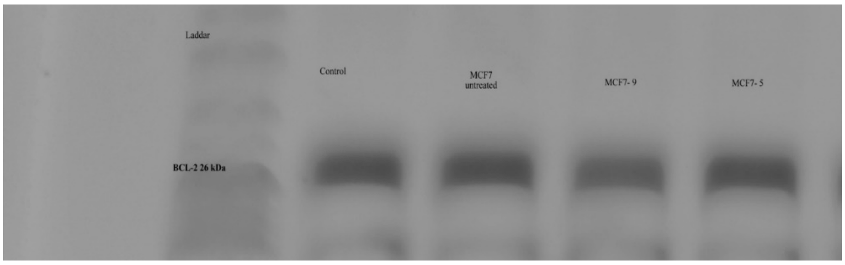

Figure S35. Uncropped western blot showing Bcl-2 (26 kDa) expression in control breast cells, untreated MCF-7 cells, and MCF-7 cells treated with compounds 5 and 9.

In this representative blot, treatment with compound 9 shows decreased Bcl-2 expression relative to untreated MCF-7 cells.
